# Deep Eutectic Solvent-Mediated Interfacial Structuring Controls Dye Adsorption and Photocatalytic Kinetics at TiO_2_–Water Interfaces

**DOI:** 10.1021/acsomega.6c03081

**Published:** 2026-07-25

**Authors:** Bruna L. Kuhn, Jean C. B. Vieira, Siara Silvestri, Pedro P. Schmaedecke, Caroline R. Bender, Clarissa Piccinin Frizzo

**Affiliations:** † Department of Chemistry (Laboratório de Líquidos Iônicos e Nanomateriais-LABLINm), Federal University of Santa Maria, Santa Maria 97105-900, Brazil; ‡ Department of Environmental Engineering (Laboratório de Engenharia do Meio Ambiente (LEMA), Federal University of Santa Maria, Santa Maria 97105-900, Brazil; § Department of Chemistry (Laboratório de Estudos de Propriedades e Interações Intermoleculares - LEPIIN), Federal University of Santa Maria, Santa Maria 97105-900, Brazil

## Abstract

Deep eutectic solvents (DES) are examined as supramolecular regulators of solid–liquid interfacial organization in TiO_2_-based photocatalytic systems. Choline chloride-derived DES, using ethylene glycol, glycerol, malonic acid, *p*-toluenesulfonic acid, or urea as hydrogen-bond donors, were investigated both as physical mixtures with TiO_2_ (TiO_2_ + DES) and as DES immobilized on TiO_2_ nanoparticles (TiO_2_-DES) to govern dye adsorption and degradation under visible light. Photocatalytic kinetics follow a Langmuir–Hinshelwood model, with apparent rate constants increasing by up to 17-fold relative to pristine TiO_2_. DES with HBDs as polyol- and organic acid-derived (ChCl:Ethyl, ChCl:Gly, and ChCl:Mal.Ac.) showed higher photocatalytic activity than ChCl:TsOH and ChCl:Urea. This enhancement may result from productive surface interactions that favor electron transfer and ROS generation, while the urea- and TsOH-based coatings likely form more strongly coordinating interfacial layers. The immobilized DES [TiO_2_-DES] generally reduces or maintains activity compared with TiO_2_ + DES. Surface plasmon resonance measurements reveal weak and reversible DES–TiO_2_ interactions, excluding permanent surface modification as the dominant mechanism. 1HH NMR spectroscopy demonstrates systematic upfield shifts of methylene blue resonances with increasing DES concentration, evidencing disruption of π–π stacking and stabilization of monomeric species in solution. The markedly stronger catalytic enhancement observed for methylene blue compared with methyl orange, which showed limited responsiveness to presence of DES (evidenced by ^1^H NMR) and is consistent with previously reported photocatalytic reports, highlights the decisive role of dye molecular structure in DES-mediated interfacial modulation.

## Graphical abstract

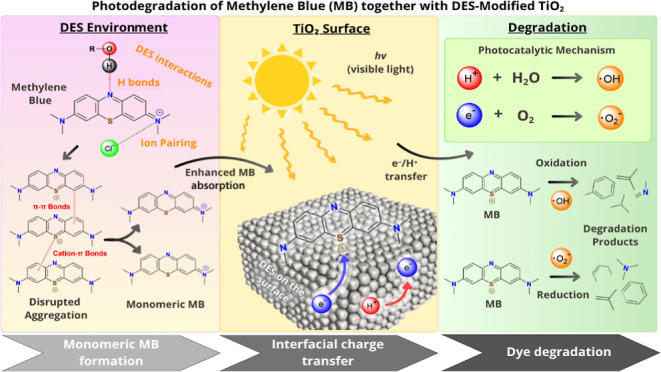

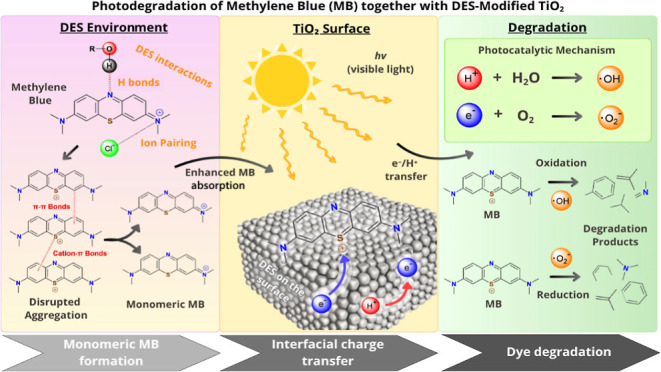

## 1 Introduction

Heterogeneous photocatalysis at oxide-liquid interfaces is fundamentally governed by interfacial thermodynamics rather than solely by semiconductor band energetics.^1–3^ For titanium dioxide (TiO_2_), reaction rates in aqueous dye degradation systems depend directly on adsorption equilibria, surface coverage, and molecular organization within the electrical double layer.^1,4,5^ Under Langmuir–Hinshelwood conditions, the apparent rate constant reflects both intrinsic surface reactivity and adsorption thermodynamics.^2,4^

Cationic aromatic dyes such as methylene blue (MB) display concentration-dependent π–π stacking governed by excitonic interactions and aggregate formation.^6,7^ Aggregation modifies electronic structure, adsorption affinity, and the likelihood of interfacial charge transfer.^3,8^ Because efficient electron injection into TiO_2_ surface states requires specific adsorption geometries, shifts in monomer–aggregate equilibria can significantly affect photocatalytic performance. Thus, modulation of supramolecular organization in the liquid phase represents a thermodynamic lever for regulating heterogeneous kinetics.

Deep eutectic solvents (DES), first introduced as hydrogen-bonded ionic mixtures by Abbott et al.,^9^ exhibit pronounced ion pairing, extensive hydrogen-bond networks, and nanoscale structuring.^10–12^ These solvents have been widely employed in the pretreatment of lignocellulosic biomass, the dissolution and modification of biopolymers, and the development of sustainable composites.^13^ A pivotal aspect of their application is the capacity for molecular restructuring, which significantly alters the supramolecular organization of complex materials. This restructuring effect is primarily driven by the ability of DES to disrupt and reorganize the intricate hydrogen-bonding networks within different substrates, facilitating processes such as swelling, fibrillation, catalysis, controlled depolymerization, and photocatalytic degradation.^2,3,13^ In addition, DES derived from choline chloride is low-cost, easy to prepare, environmentally friendly, and exhibits exceptional solvation properties for a wide range of polar and nonpolar compounds.

Spectroscopic and interfacial studies of ionic liquids demonstrate strong organization near solid surfaces, including restructuring of the electrical double layer and ion-specific adsorption phenomena.^10,14^ These characteristics suggest that DES can influence photocatalytic systems not only through bulk solvent effects but also by reorganizing the interfacial microenvironment.

Recent investigations highlight that DES-mediated synthesis can reorganize the electronic band structure of semiconductors, such as TiO_2_ and g-C_3_N_4_, by facilitating the formation of oxygen vacancies that extend light absorption into the visible and near-infrared spectra.^15,16^ This structural modulation, governed by the extensive DES hydrogen-bonding network, acts as a structure-directing medium to produce high-surface-area nanoassemblies and stabilize reactive centers, ultimately accelerating charge transfer and enhancing the efficiency of photocatalytic degradation and selective organic transformations.^17^

Two mechanistic scenarios can therefore be envisioned: (i) direct DES adsorption leading to modification of TiO_2_ surface energetics, or (ii) indirect modulation of adsorption thermodynamics via supramolecular restructuring in the diffuse interfacial region. Discriminating between these pathways is essential for elucidating how solvent structuring governs heterogeneous reactivity.

Here, we demonstrate that choline chloride-based DES (Figure 1) enhance photocatalytic degradation kinetics primarily by modulating dye aggregation rather than by strong surface functionalization. Surface plasmon resonance reveals weak, reversible interactions between DES and TiO_2_, excluding permanent surface modification as the principal mechanism. In contrast, ^1^H NMR spectroscopy demonstrates systematic upfield shifts of MB resonances with increasing DES concentration, consistent with disruption of cation−π stacking and increased monomerization. This shift in aggregation equilibrium alters adsorption thermodynamics, optimizes interfacial charge-transfer geometry, and results in substantial increases in apparent rate constants. Comparative analysis with methyl orange further confirms that enhancement magnitude depends on dye structure and aggregation propensity.

**Figure 1:**
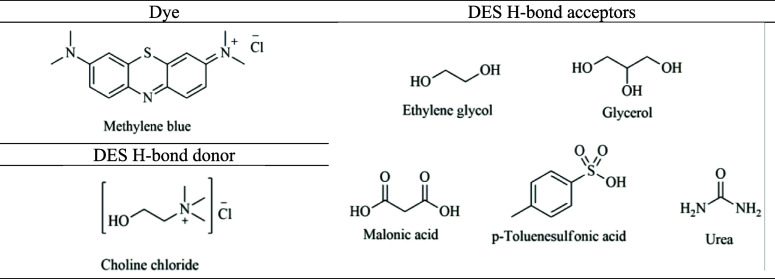
Chemical structures of methylene blue and the DES components investigated in this work.

By decoupling surface binding from liquid-phase supramolecular organization, this work establishes solvent-mediated interfacial structuring as a controllable thermodynamic parameter in adsorption-driven heterogeneous photocatalysis at oxide-liquid interfaces.

## 2 Experimental Section

### 2.1 Materials

Choline chloride (ChCl, ≥98%) was purchased from Sigma-Aldrich and dried under vacuum prior to use. Methylene blue (≥98%), methyl orange (≥98%), ethylene glycol (≥99%), glycerol (≥99%), malonic acid (≥99%), *p*-toluenesulfonic acid (≥99%), and urea (≥99%) were also obtained from Sigma-Aldrich and used as received. TiO_2_ nanoparticles (average particle size ca. 21 nm, predominantly anatase phase) were acquired from Sigma-Aldrich and used without further purification. Deionized water (Milli-Q grade) was used in all experiments.

### 2.2 DES and DES-Coated TiO_2_ Preparation

The DES and DES-coated TiO_2_ nanoparticles used in this study were prepared as reported previously.^18^ In sum, choline chloride was mixed with the respective HBD in molar ratios of 1:1 (except for urea) and 1:2. The mixtures were heated to 80 °C under magnetic stirring until homogeneous and transparent liquids formed. To prepare DES-coated TiO_2_, equimolar amounts of TiO_2_ and DES were dispersed in ethanol, heated to 40 °C, and stirred until the solvent completely evaporated. The resulting TiO_2_-DES materials were dried under vacuum at 50 °C for 72 h before use. Detailed physicochemical characterization of the DES and TiO_2_-DES systems has been reported elsewhere.^18^

### 2.3 Methylene Blue Photodegradation

Photodegradation experiments were conducted according to our previous method.^18^ Four systems were studied: (I) MB + TiO_2_, (II) MB + DES, (III) MB + TiO_2_ + DES, and (IV) MB + TiO_2_-DES. In a typical experiment, 100 mg of each component was added to 100 mL of an aqueous MB solution (20 mg L^–1^) in a cylindrical glass reactor (60 mm diameter). Prior to irradiation, the suspensions were magnetically stirred in the dark for 60 min to establish adsorption–desorption equilibrium. Irradiation was performed with a UV–vis lamp (15 W, F15/GE) operating over the 380–480 nm range. The irradiance at the solution surface was 10 W m^–2^, measured using a lux meter (MRU-201, Instrutherm). All experiments were conducted at room temperature (≈25 °C). At predetermined intervals (10, 20, 30, 45, 60, 90, and 120 min), 3 mL aliquots were withdrawn, centrifuged at 4000 rpm for 3 min to separate the catalyst, and analyzed by UV–vis spectroscopy at λ = 664 nm using a BEL Photonics 1105 spectrophotometer. All experiments were conducted in duplicate; reported values are the mean of two independent measurements.

### 2.4 Surface Plasmon Resonance Experiments

SPR measurements were carried out using a BioNavis SPR Navi 200 instrument operating in the Kretschmann configuration. Milli-Q water was employed as both solvent and mobile phase at a flow rate of 20 μL min^–1^. DES solutions were prepared at concentrations of 12.5, 25, 50, 75, and 100 μmol L^–1^ for ChCl:Mal.Ac. 1:2; 3.125, 6.25, 12.5, 25, and 50 μmol L^–1^ for ChCl:Urea 1:2; and 6.25, 12.5, 25, 50, 75, and 100 μmol L^–1^ for ChCl:TsOH 1:2 and ChCl:Ethyl 1:1. Before each measurement, the baseline was stabilized by flowing Milli-Q water over the sensor chip for 10 min. Next, 250 μL of DES solution was injected and the SPR response was recorded in real time. Between measurements, the sensor surface was regenerated by sequential injection of 250 μL of sodium dodecyl sulfate solution followed by 250 μL of isopropyl alcohol.

### 2.5 Nuclear Magnetic Resonance Experiments

^1^H NMR spectra were acquired using a Bruker Avance III spectrometer operating at 600.13 MHz for ^1^H and 150.32 MHz for ^13^C. Solutions were prepared by dissolving the appropriate amounts of dye and DES in D_2_O (1 mL) to achieve a fixed dye concentration of 7.3 mM and variable DES concentrations of 3.65, 7.3, 14.6, and 29.2 mM. Samples were transferred to 5 mm NMR tubes and measured at 25 °C. Chemical shifts were referenced to the residual solvent signal.

### 2.6 Fourier-Transform Infrared Spectroscopy (FTIR) Experiments

FTIR experiments were obtained with a Vertex 70 (Bruker) spectrometer equipped with an ATR accessory. Sixty-four scans were recorded in the range of 30–4000 cm^–^1 at a resolution of 4 cm^–^1. The tests were prepared with both the pure DES and its counterpart on the surface of TiO_2_. This procedure was performed for all deep eutectic solvents. Data analysis was accomplished using OriginPro v. 10.1 (OriginLab Corporation).

### 2.7 Raman Spectroscopy Experiments

Raman spectroscopy measurements of all compounds were performed on a Bruker Senterra spectrometer, using a 785 nm laser equipped with 20× and 50× Olympus objective lenses, in the spectral window of 3500 to 50 cm^–1^. For this analysis, tests were prepared with both the pure DES and TiO_2_-DES. Data analysis was accomplished using OriginPro v. 10.1 (OriginLab Corporation).

### 2.8 Zeta Potential Experiments

Zeta potential was evaluated using Zetasizer Lab equipment (Zetasizer Lab, Malvern Instruments), with measurements taken via mixed-mode light scattering with phase analysis (M3-PALS). The samples were prepared by diluting the previously prepared DES or TiO_2_-DES on a 1:1 ethanol and water solution, at a concentration of 50 mM. Data analysis was achieved using Zetasizer integrated software v. 3.3.1.5 (ZS Xplorer).

## 3 Results and Discussion

The photocatalytic degradation of MB was investigated by isolating the intrinsic activity of TiO_2_ from interfacial effects introduced by DES. Pristine TiO_2_ induced a 61% decrease in MB absorbance after 120 min under UV-violet-blue irradiation, whereas DES alone resulted in no measurable degradation (Figure S1). These control experiments confirm that DES are not photoactive species, but rather modify the TiO_2_-solution interface. When DES and TiO_2_ were introduced simultaneously into the MB solution ([TiO_2_ + DES]), the kinetic response changed substantially (Figure 2). Two distinct kinetic regimes emerged, demonstrating that DES composition determines the thermodynamic and structural state of the TiO_2_-MB interface.

**Figure 2:**
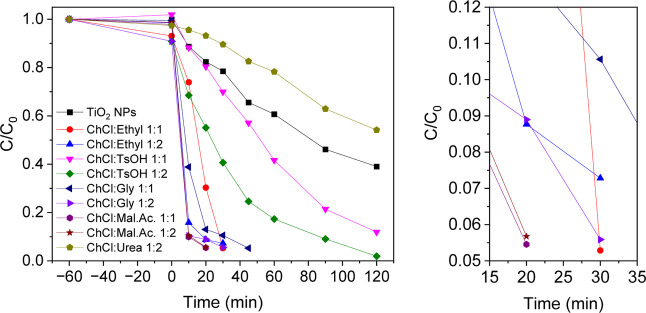
Time-dependent normalized concentration of methylene blue (*C*/*C*_0_) during photocatalysis using the TiO_2_/DES catalytic system. The right panel shows an expanded view of the left panel.

A first group of DES (ChCl:Ethyl 1:1, ChCl:Ethyl 1:2, ChCl:Gly 1:2, ChCl:Mal.Ac. 1:1, and ChCl:Mal.Ac. 1:2) induced rapid degradation, reducing MB concentration to below 8% within 30 min and completely suppressing its characteristic absorption band after 45 min. In contrast, a second group (ChCl:Urea 1:2, ChCl:TsOH 1:1, and ChCl:TsOH 1:2) exhibited substantially slower kinetics, with some systems failing to reach 92% degradation even after 120 min. ChCl:Gly 1:1 displayed intermediate behavior, leaving 5.3% residual MB after 45 min. This bifurcation cannot be attributed to simple solvent dilution, as the presence of DES components at the interface alters the intrinsic reactivity of the catalyst beyond mere concentration effects.^14,15^ Rather, it reflects the DES-dependent modulation of adsorption equilibria, interfacial charge transfer, and mass transport at the TiO_2_ surface.^15,19^ DES containing small, highly polar hydrogen-bond donors (ethylene glycol and glycerol and malonic acid) appear to stabilize adsorption configurations since they enhance electronic coupling between MB and TiO_2_, likely by inducing the formation of oxygen vacancies and surface defects that favor charge transfer, just as studied by Zhou et al.^15^ Conversely, urea- and TsOH-based DES, according to Kuhn et al.,^18^ promote competitive adsorption for active sites or excessive stabilization of MB in the bulk solution through strong intermolecular interactions, thereby limiting productive electron injection at the interface.^20^

Thus, the kinetic divergence across DES formulations reflects how hydrogen-bond and ion–dipole networks reshape the TiO_2_–MB interfacial free-energy landscape. These networks modulate the surface dipole and conduction-band alignment, shifting the driving force for charge transfer.^19,21^ In this context, the DES functions as a molecular regulator that can determines whether MB adopts geometries favorable for rapid charge injection and radical-mediated oxidation or becomes trapped in specific solvation states, such as hydration-dependent regimes which can suppress catalytic turnover.^18,22,23^

The hydrogen-bond donor (HBD) identity within the DES emerged as the principal determinant of interfacial reactivity. By modulating adsorption geometry, interfacial charge-transfer probability, and reactive oxygen species (ROS) availability, DES composition directly controls turnover frequency. Results of the residual dye concentration after photocatalytic treatment are expressed in Tables 1 and 2 as a percentage of the initial dye concentration. Therefore, lower values indicate higher degradation efficiency. The presence and composition of the DES affect the TiO_2_ surface environment and may either favor dye degradation or hinder photocatalytic activity, depending on the DES components and molar ratio.

**Table 1:** Residual Methylene Blue Concentration after Photocatalytic Treatment Using TiO_2_-DES Systems^a^

DES	residual MB (%)^a^	time (min)	DES	residual MB (%)^a^	time (min)
ChCl:Ethyl 1:1	5.3	30	ChCl:Gly 1:1	5.3	45
ChCl:Ethyl 1:2	7.3	30	ChCl:Gly 1:2	5.6	30
ChCl:TsOH 1:1	12	120	ChCl:Mal.Ac.1:1	5.4	20
ChCl:TsOH 1:2	1.9	120	ChCl:Mal.Ac.1:2	5.7	20
ChCl:Urea 1:2	54	120	TiO2 pristine	61	120

a Residual dye concentration relative to the initial methylene blue concentration after photocatalytic treatment. Lower values indicate higher photocatalytic degradation efficiency.

**Table 2:** Residual Methylene Blue Concentration After Photocatalytic Treatment Using TiO_2_+DES Systems

DES	residual MB (%)^a^	time (min)	DES	residual MB (%)^a^	time (min)
ChCl:Ethyl 1:1	10	30	ChCl:Gly 1:1	5	30
	3	45		1	45
ChCl:Ethyl 1:2	12	30	ChCl:Gly 1:2	2	30
	5	45	ChCl:Mal.Ac. 1:1	12	30
ChCl:TsOH 1:1	72	120	ChCl:Mal.Ac. 1:2	3	45
ChCl:TsOH 1:2	68	120		6	30
ChCl:Urea 1:2	53	120	TiO_2_ (pristine)	61	45

a Residual dye concentration relative to the initial methylene blue concentration; lower values indicate higher degradation.

[TiO_2_ + ChCl:Mal.Ac.] (1:1 and 1:2) exhibited the highest activity, surpassing 94% degradation within 20 min [TiO_2_ + ChCl:Ethyl] (both ratios) and [TiO_2_ + ChCl:Gly 1:2] showed comparable but slightly slower kinetics. In sharp contrast, [TiO_2_ + ChCl:Urea 1:2] failed to achieve 50% degradation after 120 min, indicating severe suppression of catalytic turnover (Figure 3) Organic acid- and polyol-based DES, according to Liu et al.,^24^ likely generate dynamic hydrogen-bond networks that reorganize MB solvation without impeding access to reactive TiO_2_ sites, thereby promoting electron injection and the formation of reactive oxygen species (ROS).^15,24^ Strongly coordinating donors (urea, TsOH), however, appeared to shift adsorption equilibria toward nonproductive states or partially occupy surface hydroxyl groups, effectively acting as chemical modifiers that can reduce charge-transfer efficiency.^25^ By competing for active surface sites or altering the local dielectric environment, these coordinating components can hinder the productive interaction between the dye and the catalyst interface.^18^ Our results evidenced that the HBD identity operated as a molecular-level regulator of the TiO_2_-MB interface, dictating whether interfacial structuring enhances or inhibits photocatalytic efficiency.

**Figure 3:**
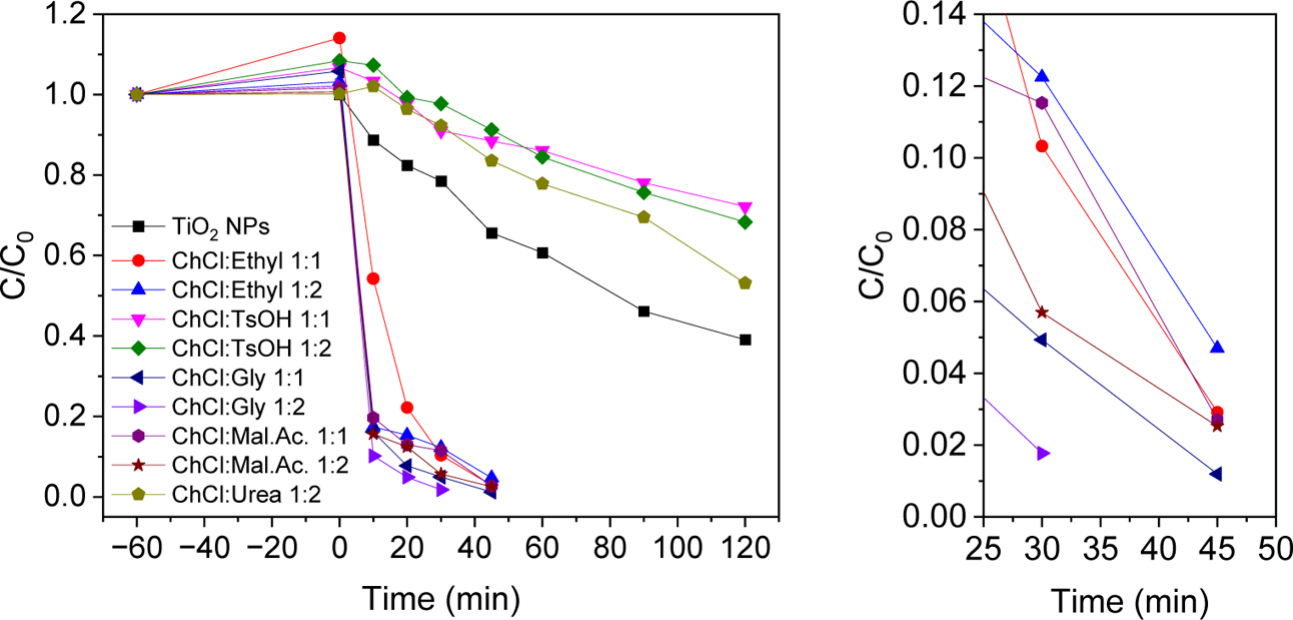
Time-dependent normalized concentration of methylene blue (MB, *C*/*C*_0_) in the TiO_2_-DES catalytic system. The right panel shows an expanded view of the left panel.

The behavior of DES-coated TiO_2_ nanoparticles ([TiO_2_–DES]) further corroborates the interfacial regulatory role of the eutectic phase.^18,20^ As in the mixed systems, distinct kinetic regimes were observed: coatings derived from polyols and organic acids (ChCl:Ethyl, ChCl:Gly, and ChCl:Mal.Ac.) exhibited enhanced photocatalytic activity compared to those based on ChCl:TsOH and ChCl:Urea. While polyol- and acid–based coatings preserve productive surface interactions may facilitate electron transfer and ROS generation through the formation of surface defects,^15,24^ urea- and TsOH-based coatings create more strongly coordinating interfacial layers. These layers can partially shield reactive surface sites and restrict MB access, effectively acting as competitive inhibitors for the photocatalytic process.^18,25^ In this manner, DES coatings do not merely alter wettability; they reshape the electronic and solvation environment at the catalyst boundary.^21^

Table 2 summarizes MB degradation for coated systems. Compared to [TiO_2_ + DES], immobilized DES [TiO_2_-DES] generally reduces or maintains activity for most formulations, indicating that surface confinement alters interfacial dynamics in a DES composition-dependent manner. Most coated systems yielded less than 92% degradation after 30 min, while mixed systems achieved more rapid MB degradation. The most active coating was [TiO_2_–ChCl:Gly 1:2], achieving >98% removal within 30 min. This exceptional performance aligns with reports that glycerol-based DES facilitate the formation of surface oxygen vacancies and enhance the density of active states, which are critical for rapid photocatalytic turnover.^15^ In contrast, [TiO_2_–ChCl:TsOH] (both ratios) failed to achieve even 50% degradation after 120 min, while [TiO_2_–ChCl:Urea 1:2] mirrored its dispersed counterpart, suggesting an intrinsic suppression of interfacial reactivity. These trends corroborate findings that strongly coordinating components, such as urea or acidic donors, can act as surface poisons or chemical modifiers that block active sites rather than merely solvating the dye.^18,25^

The DES composition plays an important role in modulating the photocatalytic performance of TiO_2_, either by improving the interaction between the dye and the catalyst surface or, in some cases, by partially hindering the access of the dye molecules to photoactive TiO_2_ sites. By comparing TiO_2_ + DES and TiO_2_-DES, one can observe the importance of dynamic interfacial organization. In suspension, DES-TiO_2_ interactions remained labile, allowing continuous reorganization of interface and charge-transfer pathways. Surface confinement imposes a more static architecture, which may restrict mass transport and radical accessibility. Moreover, the apparent rate constants were determined using the Langmuir–Hinshelwood model (eq 1).^6^

1 $$\ln\left(\frac{C}{C_{0}}\right)=-kt$$

*k* is the photodegradation rate constant, *C* is the concentration value of dye at time *t*, and *C*_0_ is the concentration of the dye at the beginning of the reaction. *k* (min^–1^), was obtained from the linear fit of ln­(*C*/*C*_0t_) versus irradiation time (*t*), likewise, calculated *k* values and correlation coefficients (*r*). Under these conditions, MB degradation follows pseudo-first-order kinetics. The k values and correlation coefficients are summarized in Table 3.

**Table 3:** Photodegradation Rate Constants for the Studied Catalytic Systems^a^

DES	*k* [TiO_2_ + DES]	*k*/*k*_0_	k [TiO_2_–DES]	*k*/*k*_0_
ChCl:Ethyl 1:1	0.095	11.88	0.082	10.25
ChCl:Ethyl 1:2	0.084	10.50	0.059	7.38
ChCl:TsOH 1:1	0.018	2.25	0.003	0.38
ChCl:TsOH 1:2	0.030	3.75	0.004	0.50
ChCl:Gly 1:1	0.064	8.00	0.092	11.50
ChCl:Gly 1:2	0.085	10.63	0.129	16.13
ChCl:Mal.Ac. 1:1	0.144	18.00	0.071	8.88
ChCl:Mal.Ac. 1:2	0.143	17.88	0.075	9.38
ChCl:Urea 1:2	0.005	0.63	0.005	0.63

a *k* of pure TiO_2_: 0.008 min^–1^ (*r* = 0.9963).

[TiO_2_ + ChCl:Mal.Ac.] exhibited the highest rate constant (∼0.14 min^–1^), representing nearly an order-of-magnitude increase relative to pristine TiO_2_ (*k* = 0.008 min^–1^). In contrast, ChCl:Urea 1:2 and TiO_2_–ChCl:TsOH showed suppressed kinetics (*k* = 0.003–0.005 min^–1^). Glycerol-based coatings uniquely outperformed their mixed counterparts (Figure 4), suggesting optimized electronic coupling with retained accessibility.

**Figure 4:**
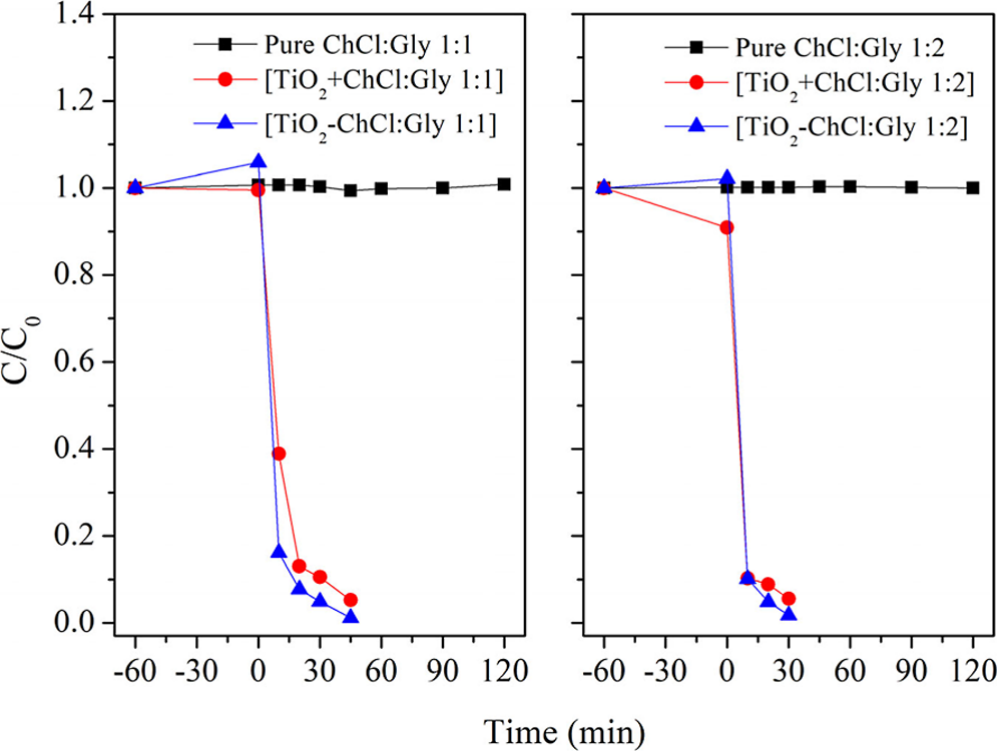
Comparison of physically mixed and ChCl:Gly-coated TiO_2_ nanoparticles.

HBD ratio exerted a secondary influence (Figures 5 and S5). According to recent reports, 1:2 formulations slightly enhance activity by strengthening hydrogen-bond networks and increasing interfacial polarity.^18,26^ This specific stoichiometry promotes clustered interfacial structuring that optimizes the local dielectric environment for charge separation.^26^ However, once this optimal organization is achieved, further HBD enrichment does not substantially affect the ROS-mediated pathway, as the fundamental mechanism of radical generation remains governed by the core DES–TiO_2_ interaction.^27^

**Figure 5:**
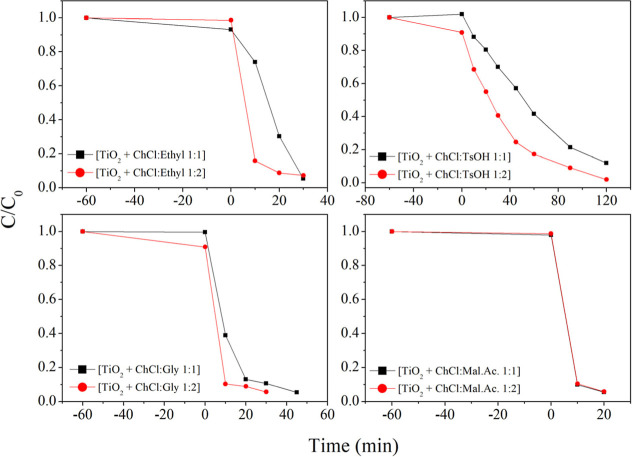
Modulation of TiO_2_ photocatalytic activity by hydrogen-bond donor fraction.

Curcumin-modified TiO_2_ ^28^ achieved 91% MB removal (*k* = 0.031 min^–1^) under deep UV irradiation. Biphenyl-porphyrin TiO_2_ ^29^ exhibited a rate constant of *k* = 0.052 min^–1^. Graphene-based systems^30,31^ similarly enhance charge separation.^32,33^ The DES-modified systems reported herein attained *k* ≈0.14 min^–1^ under violet-blue illumination without the use of dopants or sensitizers. DES function not as conventional modifiers but as adaptive interfacial solvents. In a previous study on MO,^15^ systems composed of TiO_2_ + DES (the same ones described here) showed decreased catalytic activity compared with bare TiO_2_ in the methyl orange (MO) decomposition reaction. In contrast, when TiO_2_ coated with DES was used as the catalytic system, significantly higher activities were observed. The TiO_2_ + ChCl:urea and TiO_2_ coated with ChCl:urea were evaluated in the photodegradation of methylene blue (MB). The contrasting result observed for MB could suggest that interfacial organization and adsorption processes at the catalyst surface are dye-dependent. However, the results obtained with other DES systems presented in this work indicate that this effect is not determined solely by the nature of the dye, but also by the specific interactions between the DES components and the TiO_2_ surface.

Surface plasmon resonance (SPR) was employed to investigate the interactions between DES ChCl:Mal.Ac. (1:2), ChCl:Urea (1:2), ChCl:TsOH (1:2), and ChCl:Ethyl (1:1) and a TiO_2_ (Figure 6). Measurements revealed weak, reversible interactions between DES and TiO_2_, excluding strong surface anchoring. Despite this weak interaction profile, some qualitative correlations can be identified. The DES that exhibited higher photocatalytic activity for MB degradation (ChCl:Mal.Ac. 1:2 and ChCl:Ethyl 1:1) also showed a consistent increase in the SPR signal with concentration. In contrast, ChCl:Urea (1:2) did not display a clear concentration dependence. For ChCl:TsOH (1:2), the two lowest concentrations produced higher SPR signal variation than intermediate concentrations. This trend is consistent with the photocatalytic results, in which ChCl:TsOH (1:2) exhibited relatively low activity but still outperformed ChCl:Urea (1:2) when combined with TiO_2_ nanoparticles. The SPR results indicate that DES–TiO_2_ interactions are weak and largely nonspecific. These findings support the hypothesis that the photocatalytic behavior observed in this work is governed primarily by dye-dependent interfacial effects and DES modulate the adsorption and reaction environment of MB, rather than by strong chemical attachment of DES to the TiO_2_ surface.

**Figure 6:**
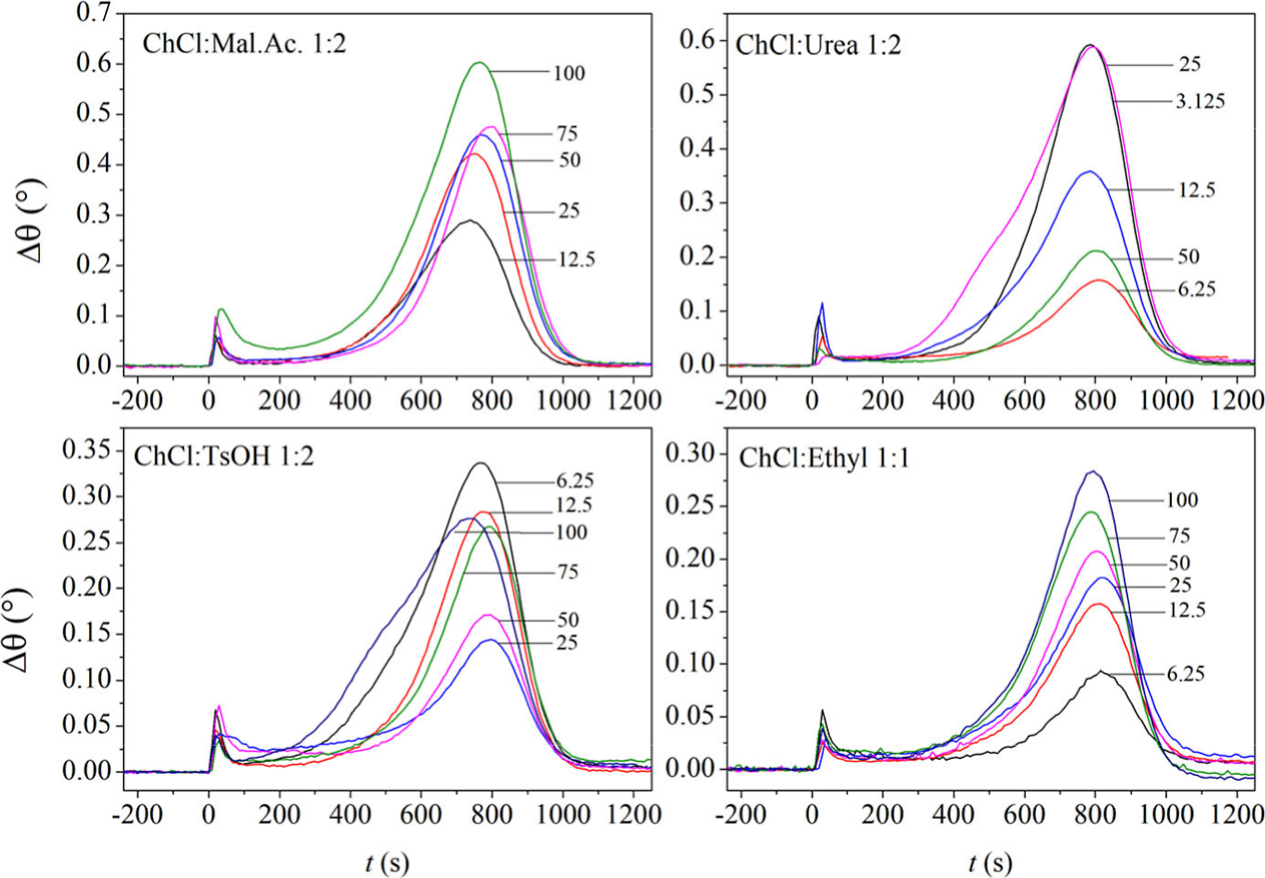
Concentration-dependent SPR sensorgrams for DES–TiO_2_ interaction.

Previous work^18^ investigated the deposition of DES onto TiO_2_ nanoparticles surace. The thermal and spectroscopic properties of DES-coated TiO_2_, compared with those of the pure materials, indicated interactions between the two components, confirming DES deposition on the TiO_2_ surface. Different choline chloride:HBD molar ratios had little effect on the thermal stability, phase transitions, crystallization, and amorphization of the mixtures. The catalytic activity of DES-coated TiO_2_ was evaluated for MO decomposition and was reduced compared with pristine TiO_2_, except for TiO_2_ coated with ChCl:malonic acid at a 1:2 molar ratio. Additional, in the present work, to address any doubt, FTIR and Raman spectroscopic analyses were performed on pure DES and DES + TiO_2_ systems. FTIR spectra of all DES-TiO_2_ samples revealed that the characteristic vibrational bands of each DES represented by O–H stretching (∼3200–3500 cm^–1^), C–H stretching (∼2900–3000 cm^–1^), and C=O stretching for malonic acid–based formulations (∼1700 cm^–1^) are fully preserved without significant frequency shifts or band broadening upon contact with TiO_2_. The absence of new absorption features or systematic spectral shifts indicates that no strong surface coordination occurs between DES components and surface sites of TiO_2_.

Raman spectra further confirmed that the anatase fingerprint bands of TiO_2_ remain unperturbed in all DES-TiO_2_ systems, indicating lower incorporation or surface-layer modification. These spectroscopic findings are fully consistent with the SPR results, which demonstrated weak and reversible DES–TiO_2_ interactions, and collectively confirm that the photocatalytic enhancement reported herein originates from DES-mediated reorganization of the interfacial microenvironment rather than from chemical absorption of DES onto the metal oxide surface.

Zeta potential measurements were also carried out for pure TiO_2_ and all DES-TiO_2_ systems. Pure TiO_2_ exhibited a zeta potential of −0.07 mV, consistent with near-neutral surface charge under the measurement conditions. Upon introduction of DES, the zeta potential values remained remarkably close to zero for most formulations, indicating that the majority of DES do not substantially alter the surface charge of TiO_2_. None of the measured values approach the thresholds typically associated with stable colloidal modification (|ζ| > 20–30 mV), confirming that DES does not induce permanent electrostatic functionalization of the metal oxide surface. These results are fully consistent with the SPR, FTIR, and Raman data, collectively supporting the conclusion that DES interactions with TiO_2_ are weak, reversible, and confined to the interfacial region rather than resulting in bulk surface absorption or chemical modification. The FT-IR, Raman, and zeta potential data can be seen in the ESI.

The chemical shifts of the MB protons progressively move upfield with increasing DES concentration, while the DES resonances remain essentially unchanged (Table 4). This systematic shielding indicates that DES interact preferentially with the dye rather than undergoing significant self-association changes. The upfield shifts suggest the presence of π–π or ion–dipole interactions between MB and the DES components. The absence of significant changes in the DES signals further indicates that DES does not undergo major structural rearrangements upon interaction with MB, supporting the hypothesis that DES primarily acts as a medium modifier rather than forming stable stoichiometric complexes with the dye.

**Table 4:**
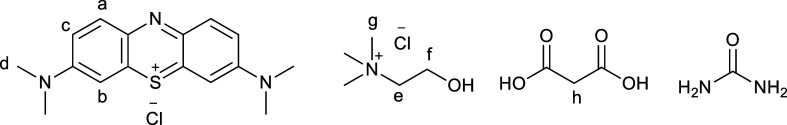
Concentration-Dependent ^1^H NMR Chemical Shifts of the DES-MB System with the Methylene Blue Concentration Fixed at 7.3 mM

MB + DES	^1^H chemical shift							
	a	b	c	D	e	f	g	h
pure MB 7.3 mM	7.41	6.88	7.11	3.19	-	-	-	-
pure ChCl:Mal.Ac 7.3 mM	-	-	-	-	3.53	4.07	3.21	3.23
MB + ChCl:Mal.Ac. 3.65 mM	7.46	6.92	7.14	3.21	3.53	4.07	3.21	3.23
MB + ChCl:Mal.Ac. 7.3 mM	7.41	6.88	7.11	3.19	3.53	4.07	3.21	3.23
MB + ChCl:Mal.Ac. 14.6 mM	7.37	6.85	7.08	3.17	3.53	4.07	3.21	3.23
MB + ChCl:Mal.Ac. 29.2 mM	7.27	6.78	7.01	3.12	3.52	4.06	3.20	3.22
Pure ChCl:Urea 7.3 mM	-	-	-	-	3.52	4.06	3.20	-
MB + ChCl:Urea 3.65 mM	7.42	6.89	7.11	3.19	3.52	4.07	3.20	-
MB + ChCl:Urea 7.3 mM	7.39	6.86	7.09	3.17	3.52	4.07	3.21	-
MB + ChCl:Urea 14.6 mM	7.36	6.84	7.08	3.16	3.52	4.07	3.21	-
MB + ChCl:Urea 29.2 mM	7.28	6.79	7.02	3.13	3.52	4.07	3.21	-

A similar trend was observed for the DES and MO (Table 5). This behavior again suggests weak, nonspecific dye–DES interactions that perturb the electronic environment of the dye without significantly affecting the DES structure. The NMR results indicate that DES interacts more strongly with the dye molecules than with the TiO_2_ surface, in agreement with the weak and nonspecific DES–TiO_2_ interactions inferred from SPR. These dye–DES interactions influence the electronic environment, solvation, and possibly the aggregation state of MB in solution, thereby affecting their accessibility and reactivity at the TiO_2_ surface. Such interactions appear to favor adsorption and interfacial reactivity, consistent with the enhanced photocatalytic activity observed for DES-modified TiO_2_ systems. Selectivity toward MB over MO further demonstrates that photocatalytic efficiency is governed by dye-DES-TiO_2_ interfacial organization, rather than by catalyst properties alone. Overall, the observed enhancement originated from DES-driven modification of the interfacial microenvironment (i.e., tuning aggregation equilibria, polarity, electrostatics, and charge-transfer accessibility) rather than chemical functionalization of TiO_2_. The ^1^H NMR analysis supports the conclusion that DES primarily act as tunable solvation and interfacial modifiers rather than forming strong, permanent interactions with TiO_2_.

**Table 5:**
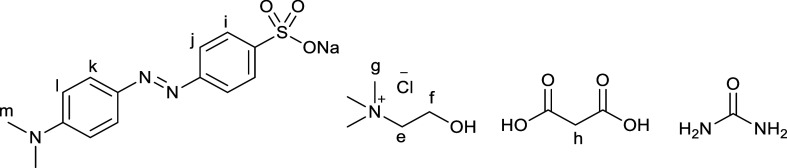
Concentration-Dependent ^1^H NMR Chemical Shifts of the MO + DES System with the Methyl Orange Concentration Fixed at 7.3 mM^a^

MO + DES	^1^H chemical shift								
	i	J	K	l	m	e	f	g	h
pure MO 7.3 mM	7.74–7.72	7.91	7.74–7.72	6.80	3.00	-	-	-	-
Pure ChCl:Urea 7.3 mM	-	-	-	-		3.52	4.06	3.20	-
MO + ChCl:Urea 3.65 mM	7.72–7.70	7.91	7.72–7.70	6.78	2.99	3.51	4.06	3.19	-
MO + ChCl:Urea 7.3 mM	7.73–7.71	7.91	7.73–7.71	6.79	2.99	3.52	4.06	3.20	-
MO + ChCl:Urea 14.6 mM	7.72–7.70	7.91	7.72–7.70	6.77	2.98	3.51	4.06	3.20	-
MO + ChCl:Urea 29.2 mM	7.70–7.68	7.91	7.70–7.68	6.75	2.97	3.52	4.06	3.20	-

a ChCl:Mal.Ac. 1:2 led to precipitation of MO when mixed in D_2_O.

Overall, the enhanced photocatalytic performance did not result from chemical modification of TiO_2_, but from DES-driven reprogramming of the interfacial microenvironment. By reshaping aggregation equilibria, local polarity, electrostatic organization, and charge-transfer accessibility at the TiO_2_-solution interface, DES redefined how the dye approaches, binds, and electronically couples to the photocatalyst (Figure 7). This work establishes DES as adaptive interfacial agents capable of tuning catalytic free-energy landscapes without doping, covalent grafting, or permanent surface functionalization. Such soft chemical control of adsorption and charge transfer provides a generalizable strategy for engineering photocatalytic interfaces via solvent design, rather than by altering the catalyst’s structure itself.

**Figure 7:**
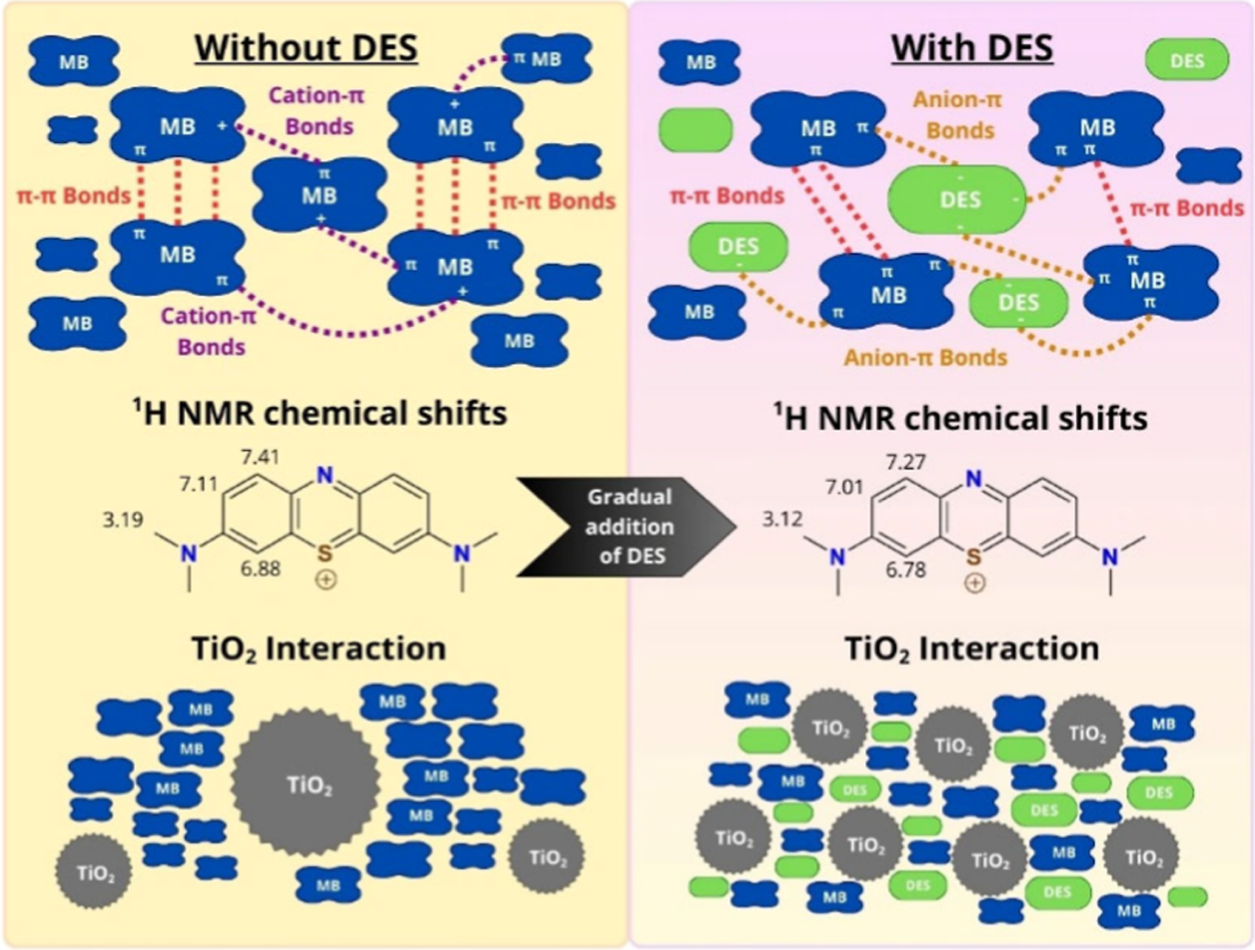
Schematic representation of the TiO_2_-DES-MB system. DES components interact preferentially with MB through hydrogen bonding, ion pairing, and disruption of π–π stacking, modulating dye aggregation and solvation.

Catalyst reusability was not evaluated in the present study. Although the TiO_2_ core is expected to be recoverable, the stability of the DES-derived organic coating under photocatalytic conditions remains uncertain, since irradiated TiO_2_ can generate highly reactive oxidizing species. Therefore, consecutive reuse cycles and postreaction characterization of the recovered catalyst are required to confirm both recyclability and surface-coating preservation.

## 4 Conclusions

This study demonstrated that DES offer a versatile and chemically soft route to control photocatalytic kinetics at TiO_2_-water interfaces via interfacial structuring rather than permanent surface modification. Choline chloride-based DES increased methylene blue degradation under visible light by up to approximately 18-fold, following Langmuir–Hinshelwood kinetics. Moreover, SPR measurements indicated weak, reversible DES-TiO_2_ interactions, thus excluding strong catalyst functionalization as the primary mechanism. Instead, ^1^H NMR showed that DES disrupts dye cation−π stacking, promoting monomerization and altering adsorption geometries that facilitate interfacial charge transfer and ROS generation. The strong dependence on hydrogen-bond donor identity and dye structure highlights photocatalysis as an emergent interfacial phenomenon governed by supramolecular organization. DES emerges as adaptive interfacial media capable of reprogramming catalytic free-energy landscapes, offering new opportunities for solvent-designed strategies in photocatalysis and oxide interface engineering.

Despite some limitations such as molecular-level information about DES–TiO_2_ interfacial organization provided by SPR and the use of photocatalytic tests under conditions that may not fully represent real wastewater matrices, the results highlight the potential of DES as tunable interfacial modifiers in photocatalytic systems. These findings provide useful insights for the development of more efficient and sustainable water-treatment technologies relevant to environmental remediation and industrial wastewater management. Future studies should explore a broader range of DES compositions, additional classes of pollutants, and complementary in situ or computational approaches to better understand interfacial effects.

## Supplementary Material



## Notes

Detailed kinetic profiles for MB photodegradation over pristine TiO_2_ NPs, neat DES, and mixed or DES-coated TiO_2_ NPs prepared with ChCl:Ethyl, ChCl:TsOH, and ChCl:Mal.Ac. are provided. The effect of HBD proportion on the activity of [TiO_2_-DES] catalytic systems is also examined, and complete ^1^H NMR spectra for all experiments are included. During the preparation of this work, the authors used SCISPACE to assist in synthesizing complex literature on TiO_2_ catalysis under DES conditions. The authors subsequently reviewed and edited the generated content as necessary and took full responsibility for the content of the published article (PDF)
