# Supplementary material for: Deep Eutectic Solvent-Mediated Interfacial Structuring Controls Dye Adsorption and Photocatalytic Kinetics at TiO2–Water Interfaces
Source: ACS Omega. 2026 Jul 25;11(31):46204–13. doi: 10.1021/acsomega.6c03081 (PMC13470898; doi:10.1021/acsomega.6c03081)
Supplement: Supplementary file 1 [file ao6c03081_si_001.pdf]

Supporting Information for

# Deep Eutectic Solvent -Mediated Interfacial Structuring Controls Dye Adsorption and Photocatalytic Kinetics at $TiO_2$–Water Interfaces

Bruna L. Kuhn[1]; Jean C. B. Vieira[1], Siara Silvestri[2], Pedro P. Schmaedecke[1], Caroline R. Bender[3] and Clarissa Piccinin Frizzo[1*]

[1]Department of Chemistry (Laboratório de Líquidos Iônicos e Nanomateriais - LABLINm), Federal University of Santa Maria, 97105–900 Santa Maria, Brazil.
[2]Department of Environmental Engineering (Laboratório de Engenharia do Meio Ambiente (LEMA), Federal University of Santa Maria, 97105–900 Santa Maria, Brazil.
[2]Department of Chemistry (Laboratório de Estudos de Propriedades e Interações Intermoleculares - LEPIIN), Federal University of Santa Maria, 97105–900 Santa Maria, Brazil.

*clarissa.frizzo@gmail.com

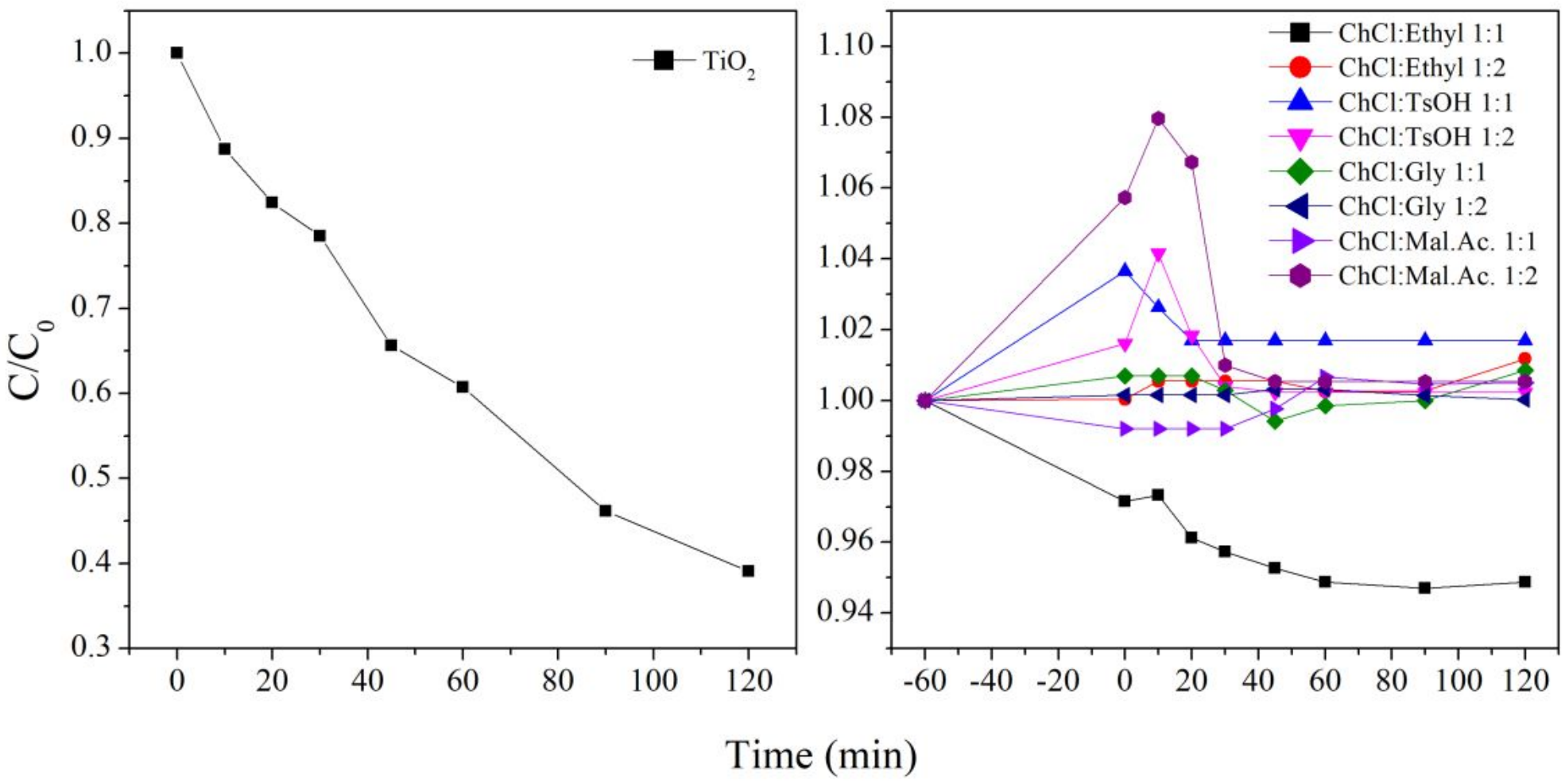


**Fig S1.** Photodegradation of MB using pure $TiO_2$ NPs or pure DES

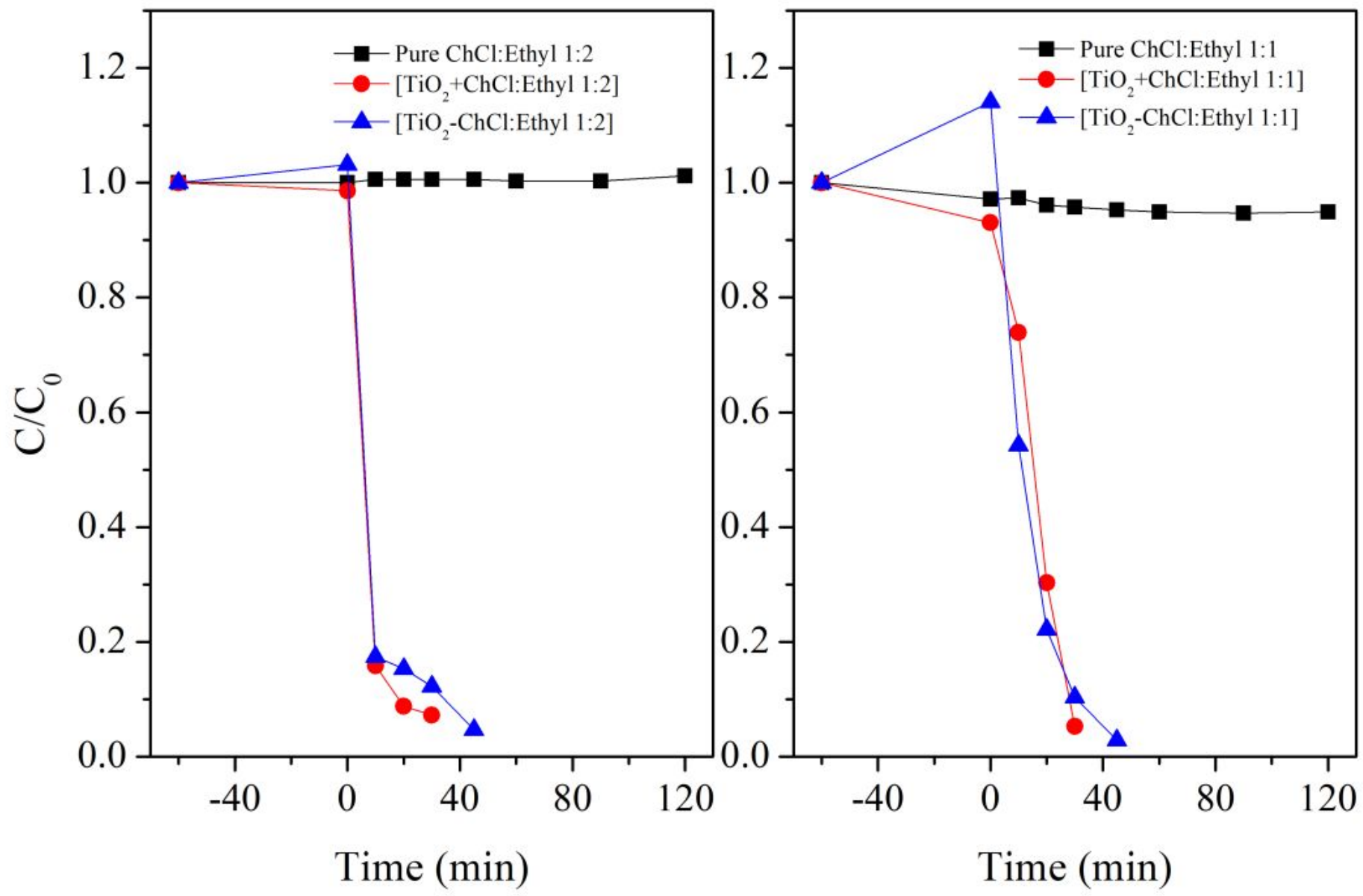


**Fig. S2** Comparison between mixed and coated $TiO_2$ NPs with ChCl:Ethyl

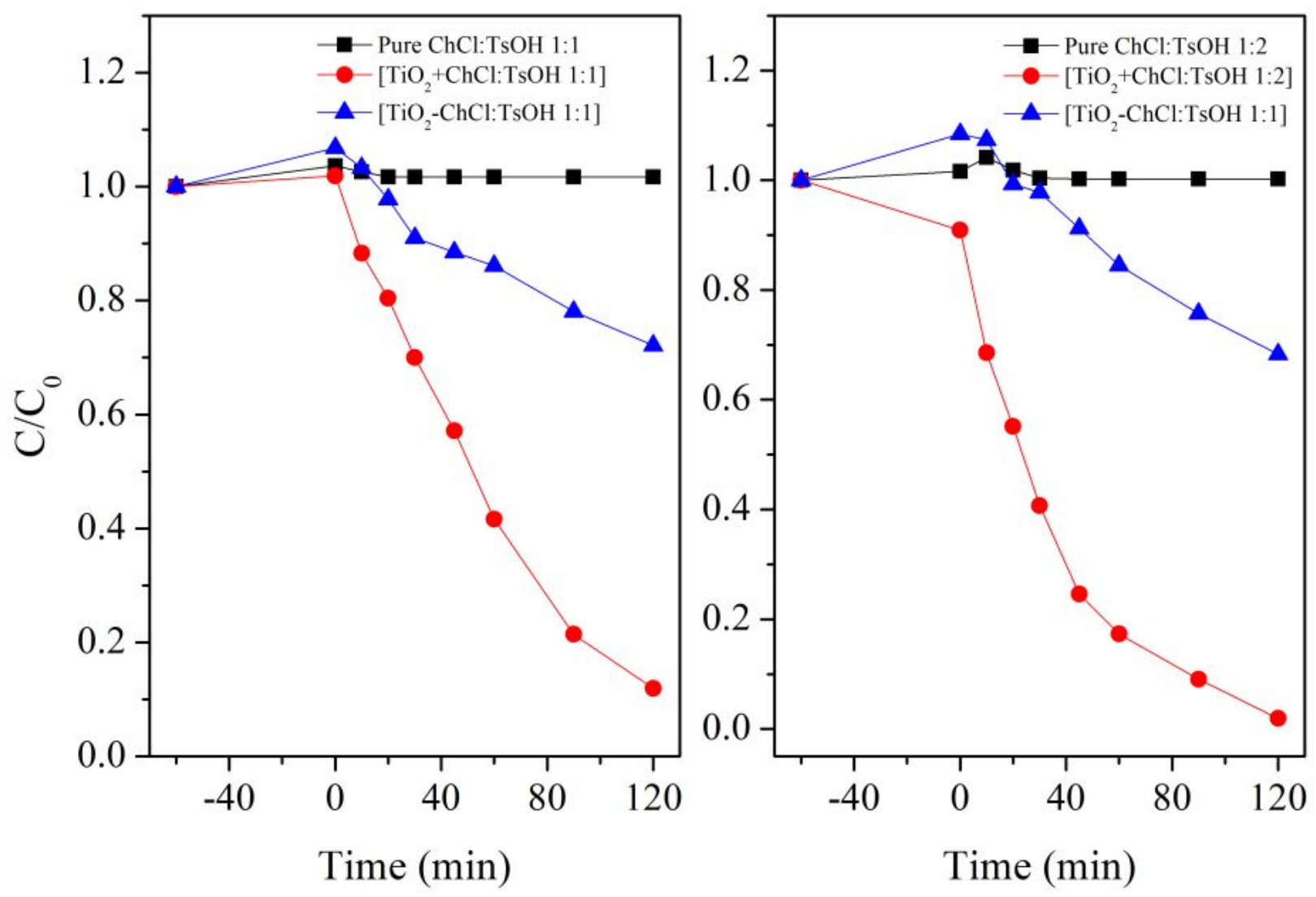


**Fig. S3** Comparison between mixed and coated $TiO_2$ NPs with ChCl:TsOH

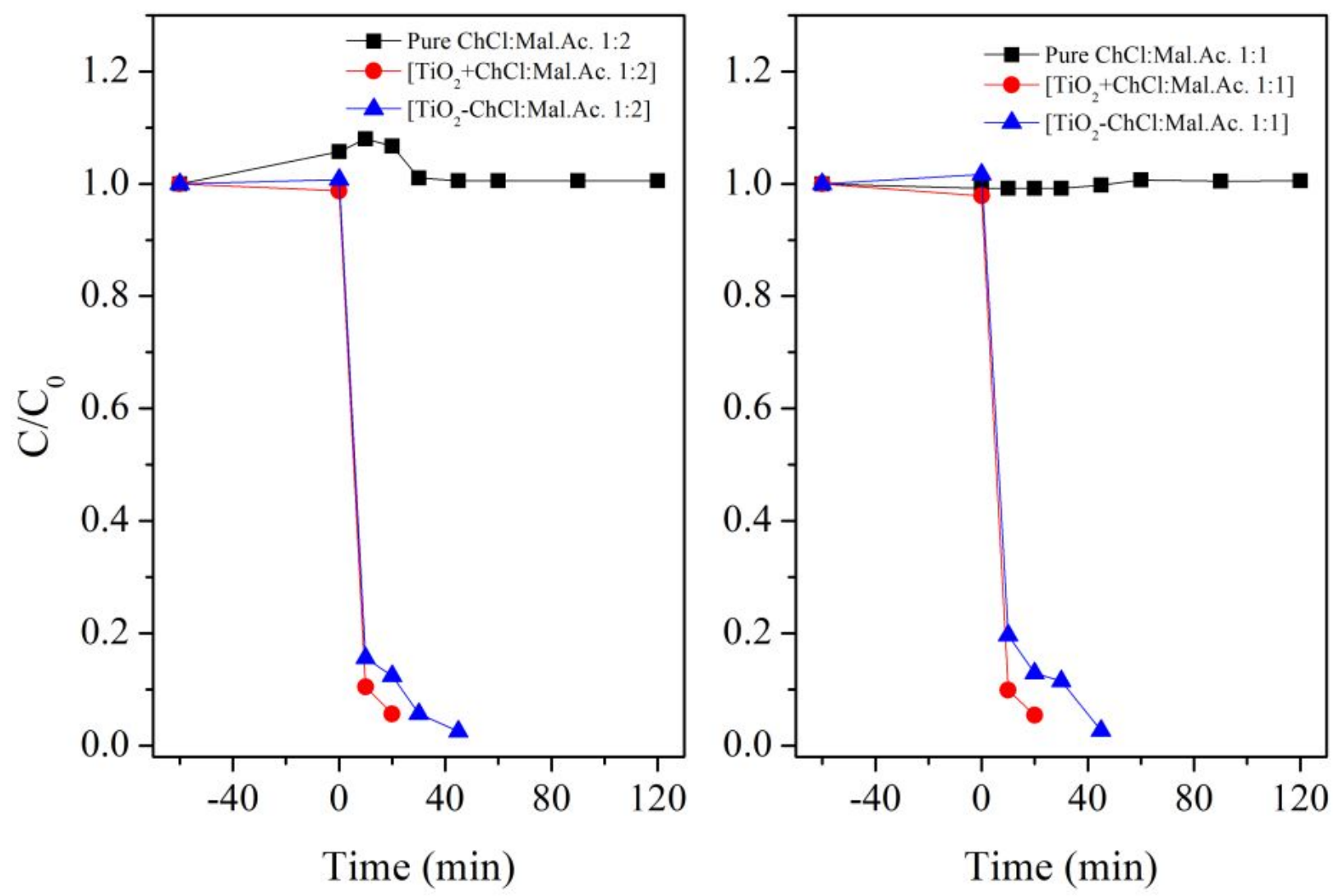


**Fig. S4** Comparison between mixed and coated $TiO_2$ NPs with ChCl:Mal.Ac.

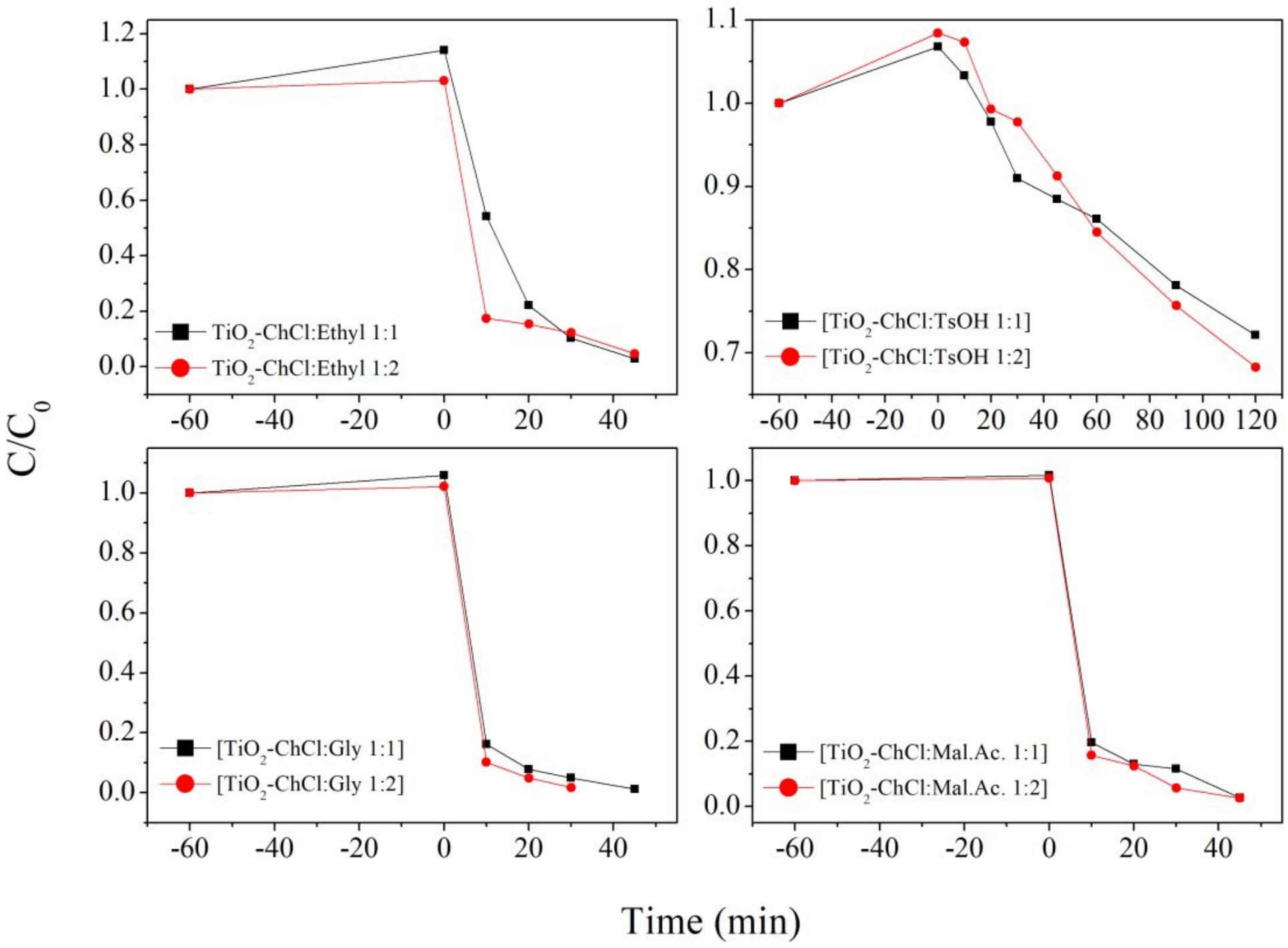


**Fig. S5** Influence of HBD proportion in DES for [$TiO_2$-DES] catalytic systems

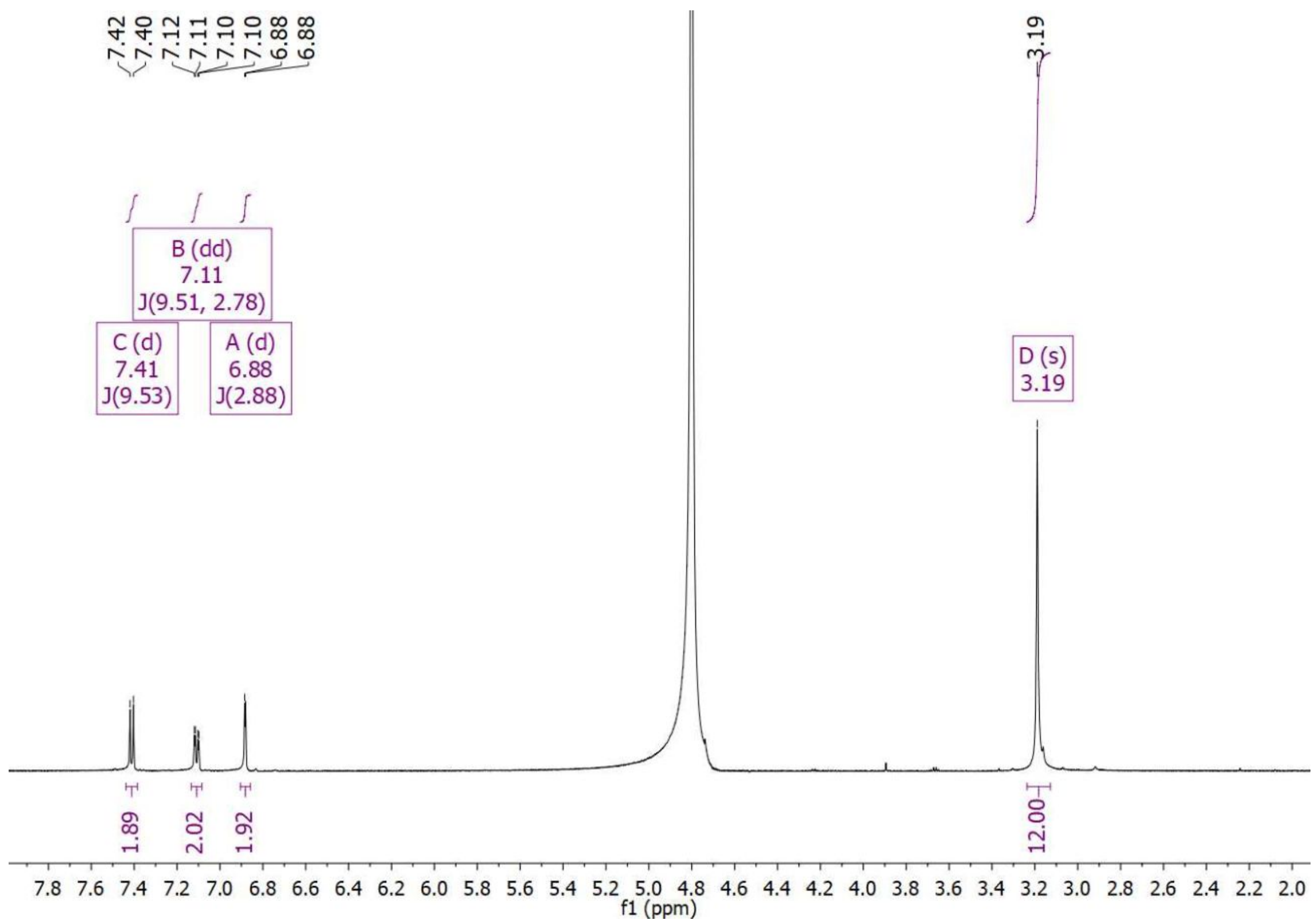


**Fig S6** $^{1}$H NMR spectrum of MB at 7.3 mM ($D_2O$, 600 MHz, 25 °C).

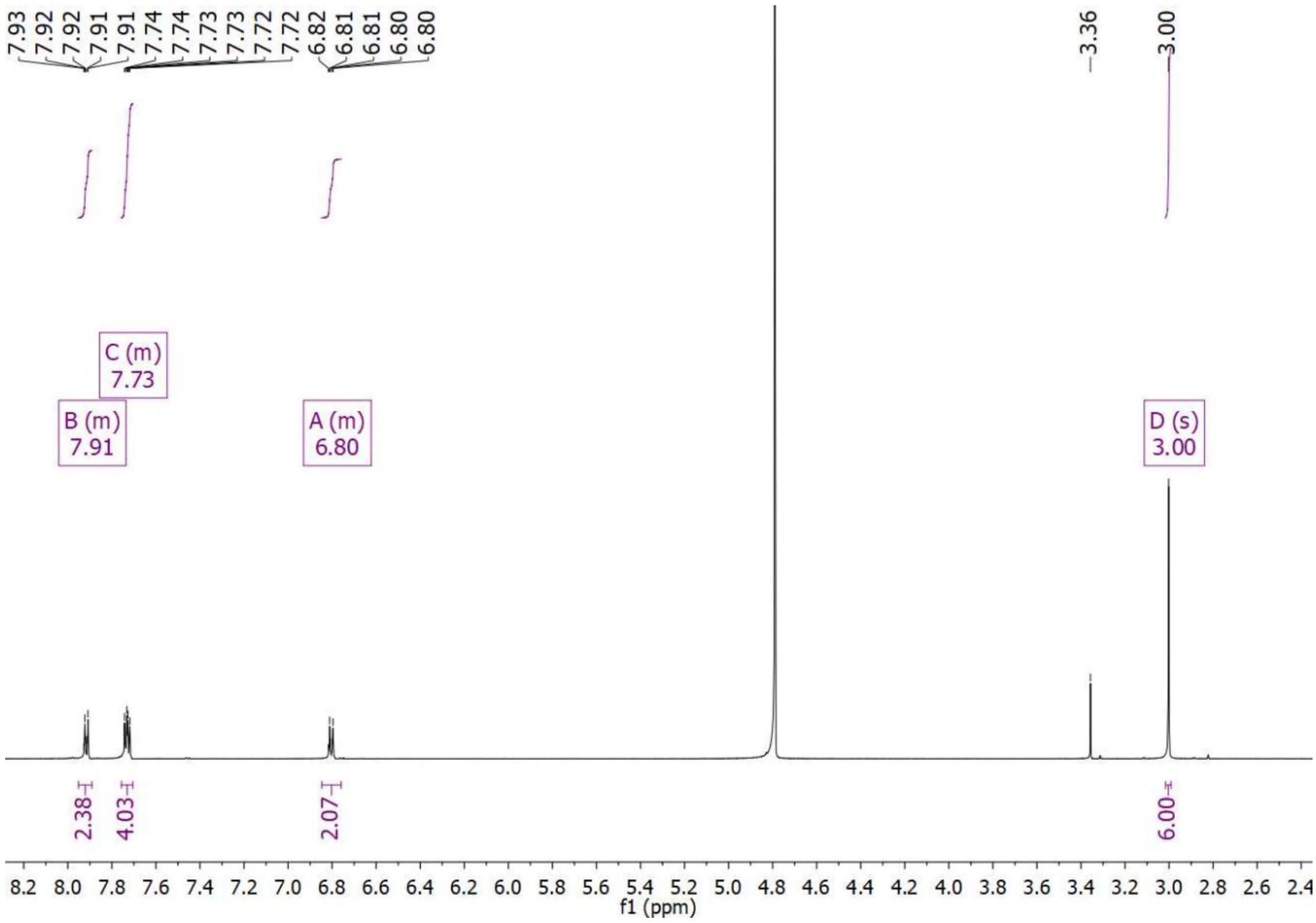


**Fig S7** $^{1}$H NMR spectrum of MO at 7.3 mM ($D_2O$, 600 MHz, 25 °C).

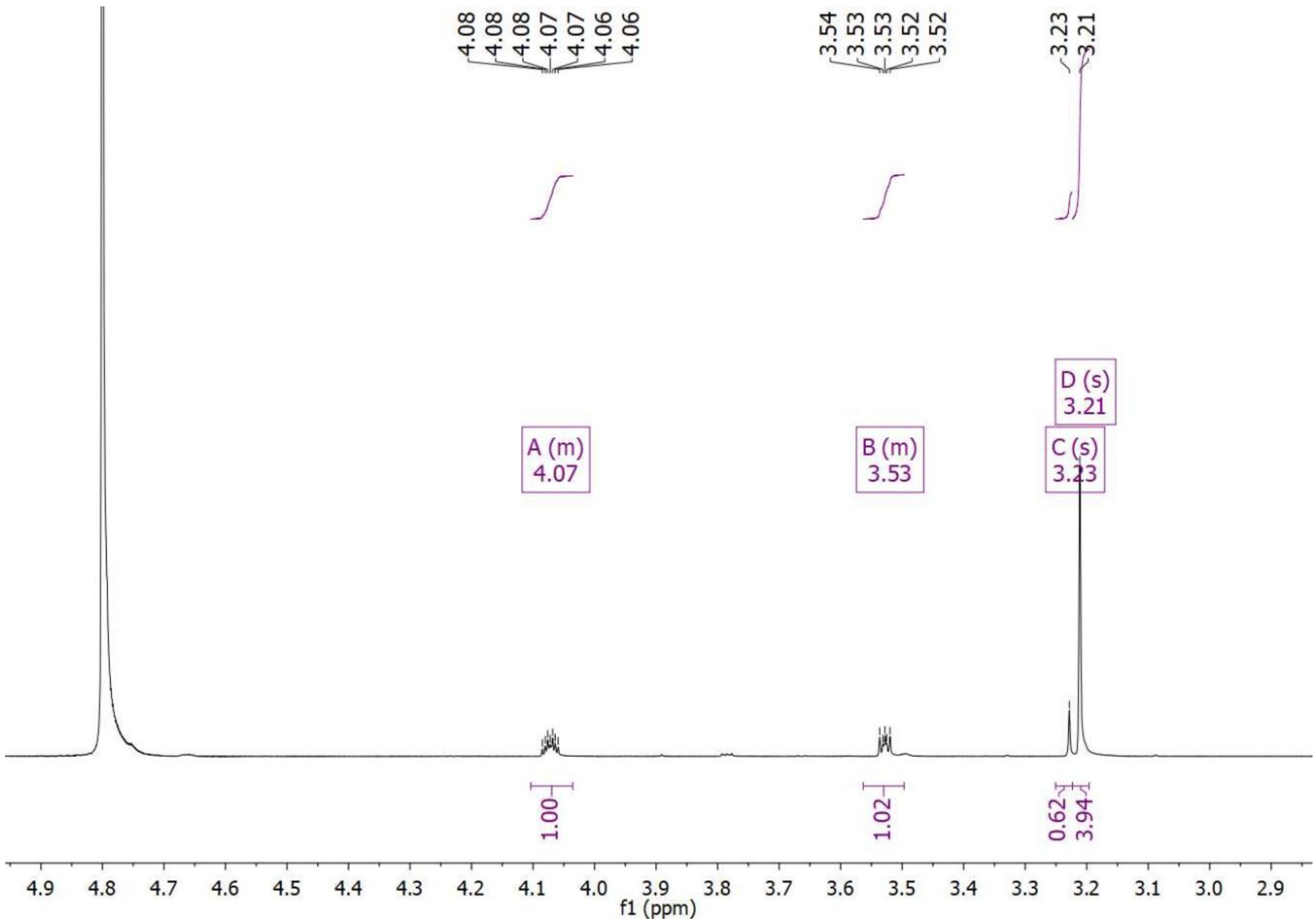


**Fig S8** $^{1}$H NMR spectrum of ChCl:Mal.Ac. 1:2 at 7.3 mM ($D_2O$, 600 MHz, 25 °C).

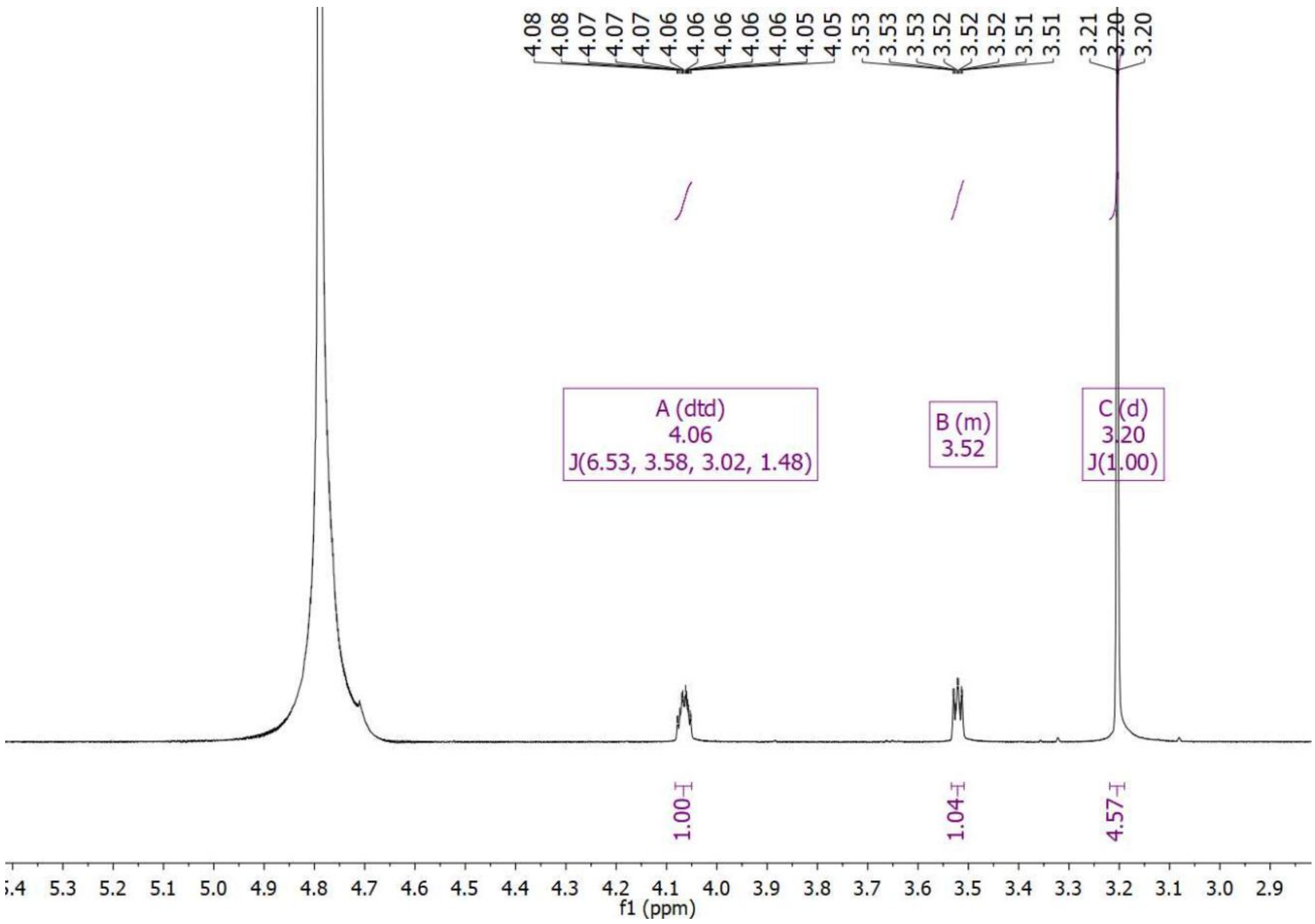


**Fig S9** $^{1}$H NMR spectrum of ChCl:Urea 1:2 at 7.3 mM ($D_2O$, 600 MHz, 25 °C).

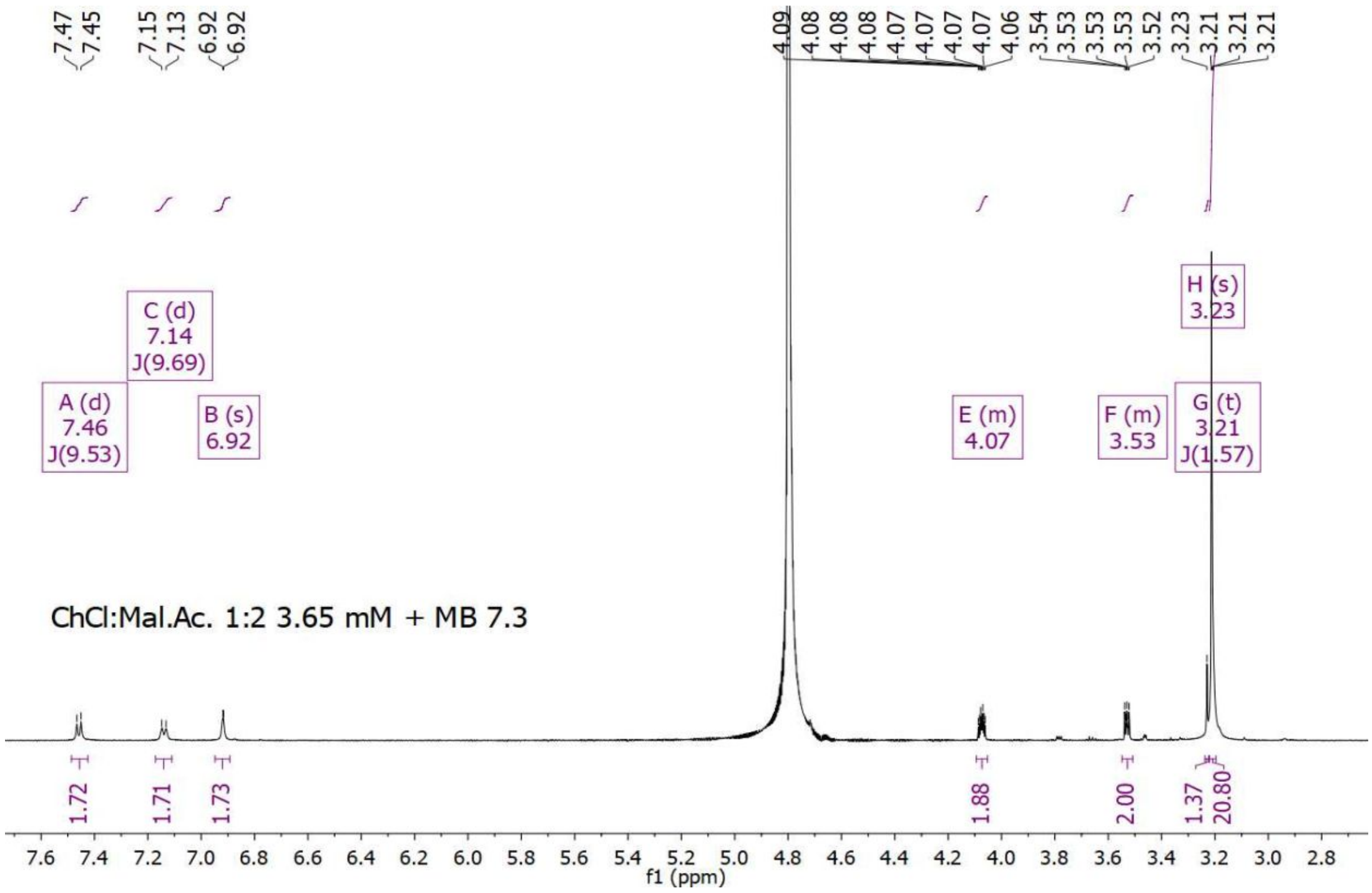


**Fig S10** $^{1}$H NMR spectrum of ChCl:Mal.Ac. 1:2 3.65 mM + MB 7.3 mM ($D_2O$, 600 MHz, 25 °C).

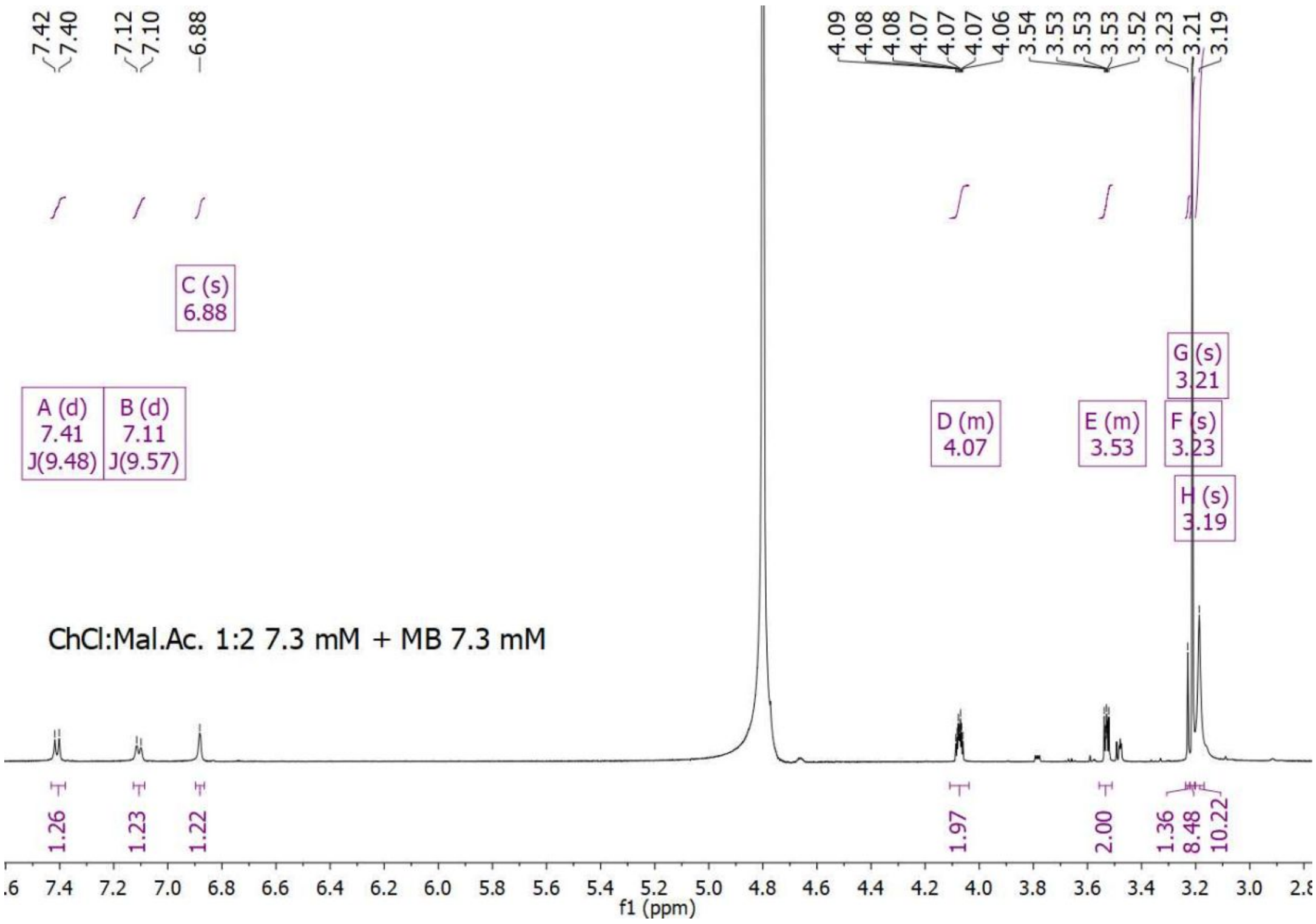


**Fig S11** $^{1}$H NMR spectrum of ChCl:Mal.Ac. 1:2 7.3 mM + MB 7.3 mM ($D_2O$, 600 MHz, 25 °C).

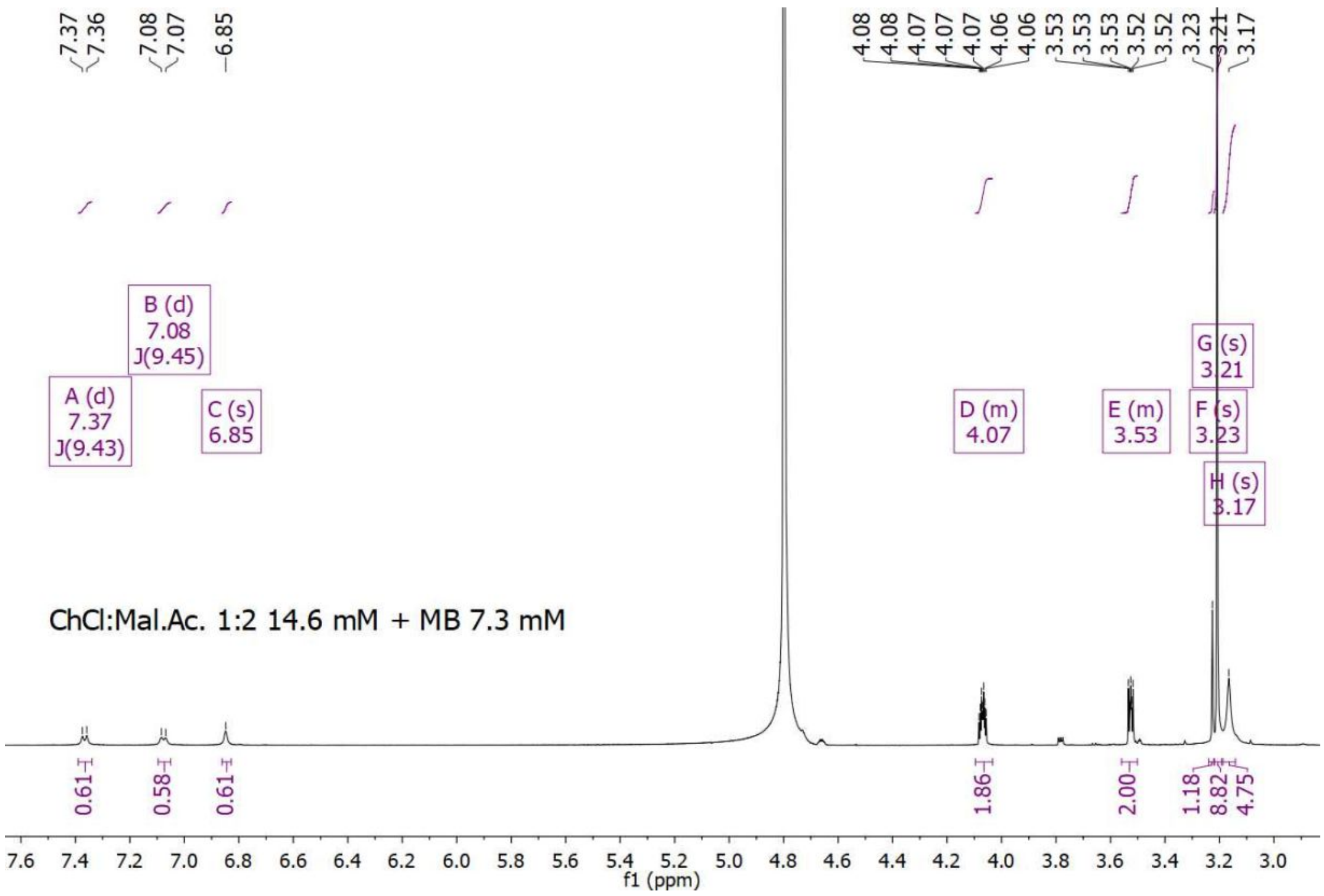


**Fig S12** $^{1}$H NMR spectrum of ChCl:Mal.Ac. 1:2 14.6 mM + MB 7.3 mM ($D_2O$, 600 MHz, 25 °C).

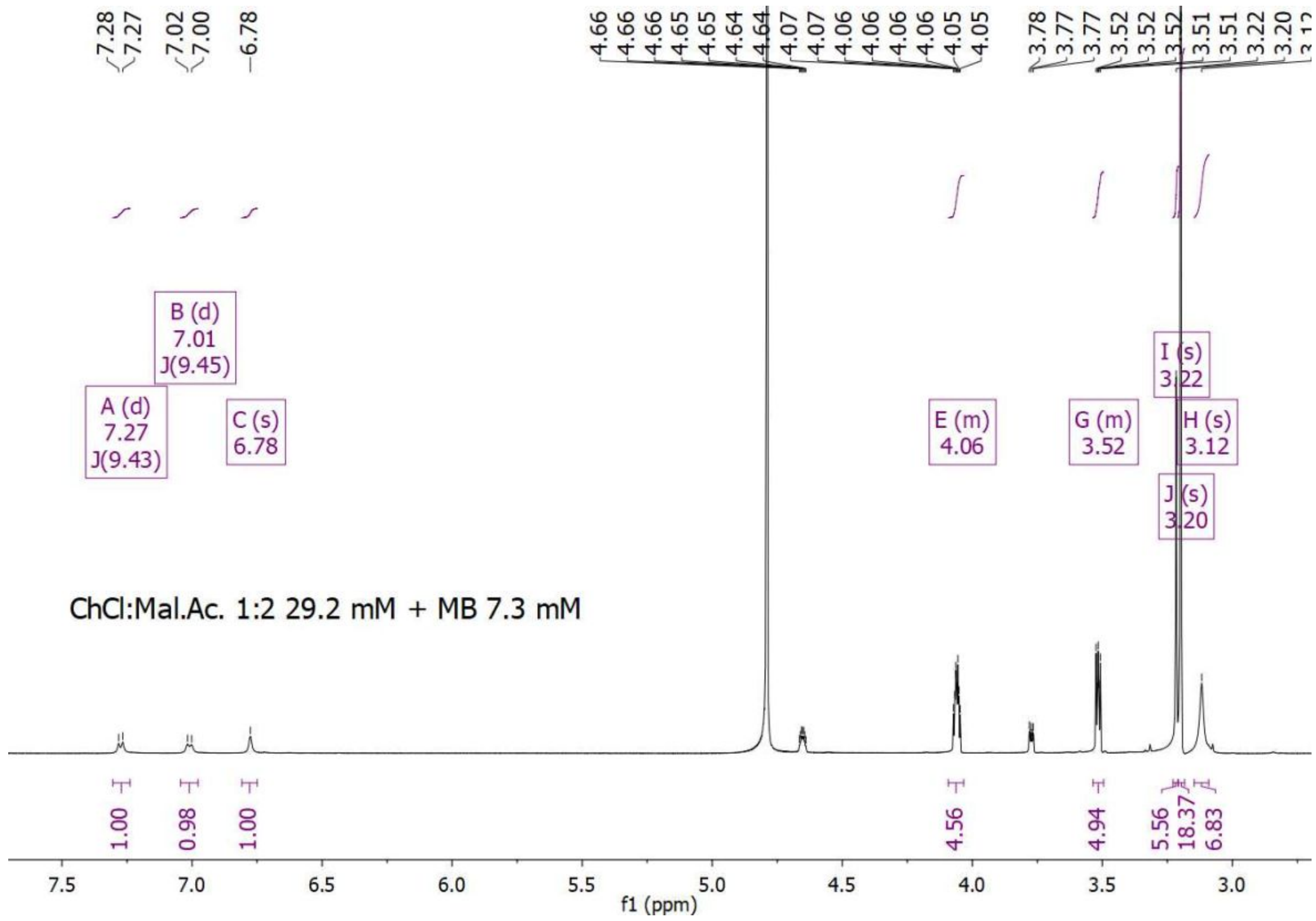


**Fig S13** $^1$H NMR spectrum of ChCl:Mal.Ac. 1:2 29.2 mM + MB 7.3 mM ($D_2O$, 600 MHz, 25 °C).

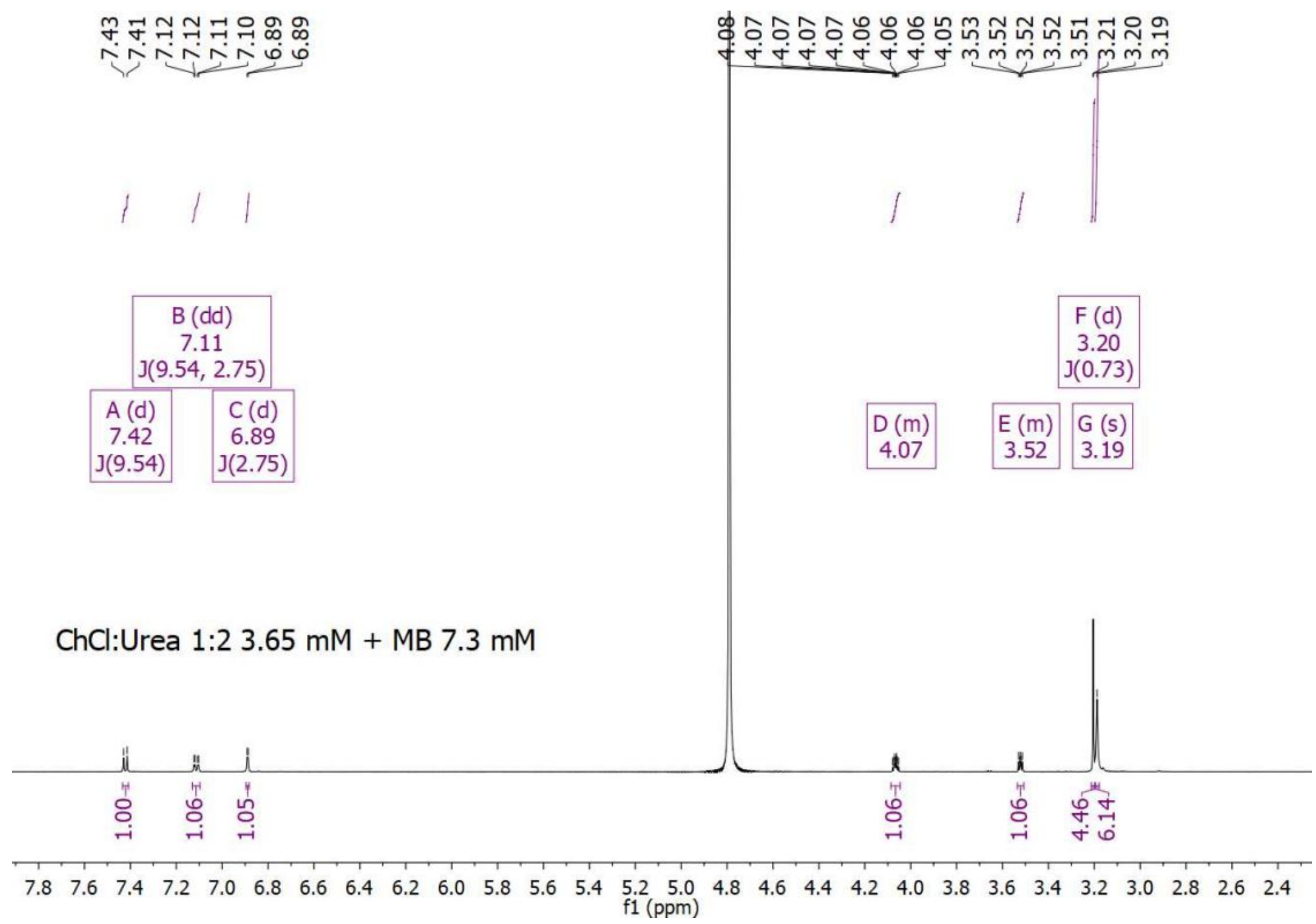


**Fig S14** $^1$H NMR spectrum of ChCl:Urea 1:2 3.65 mM + MB 7.3 mM ($D_2O$, 600 MHz, 25 °C).

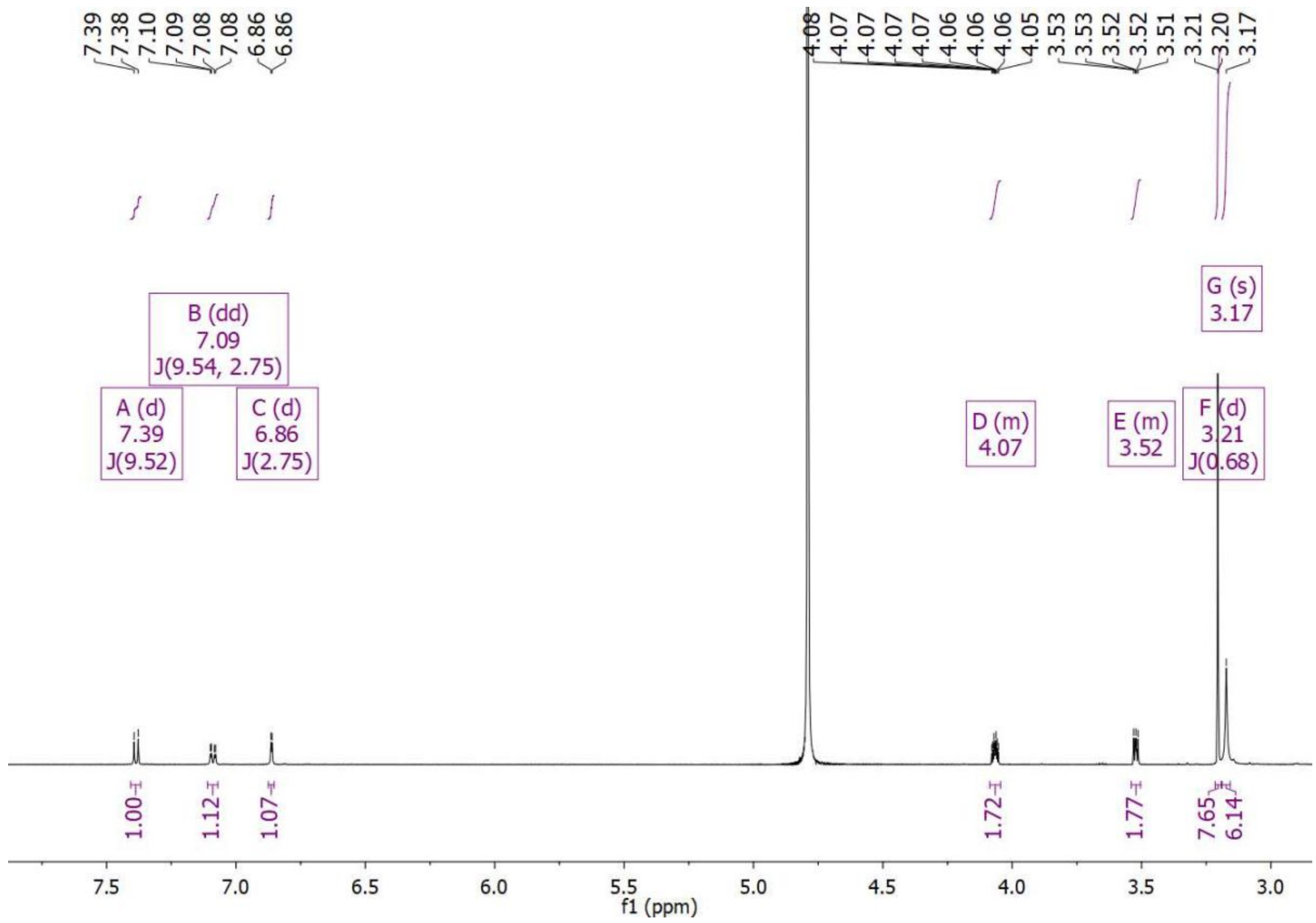


**Fig S15** $^{1}$H NMR spectrum of ChCl:Urea 1:2 7.3 mM + MB 7.3 mM ($D_2O$, 600 MHz, 25 °C).

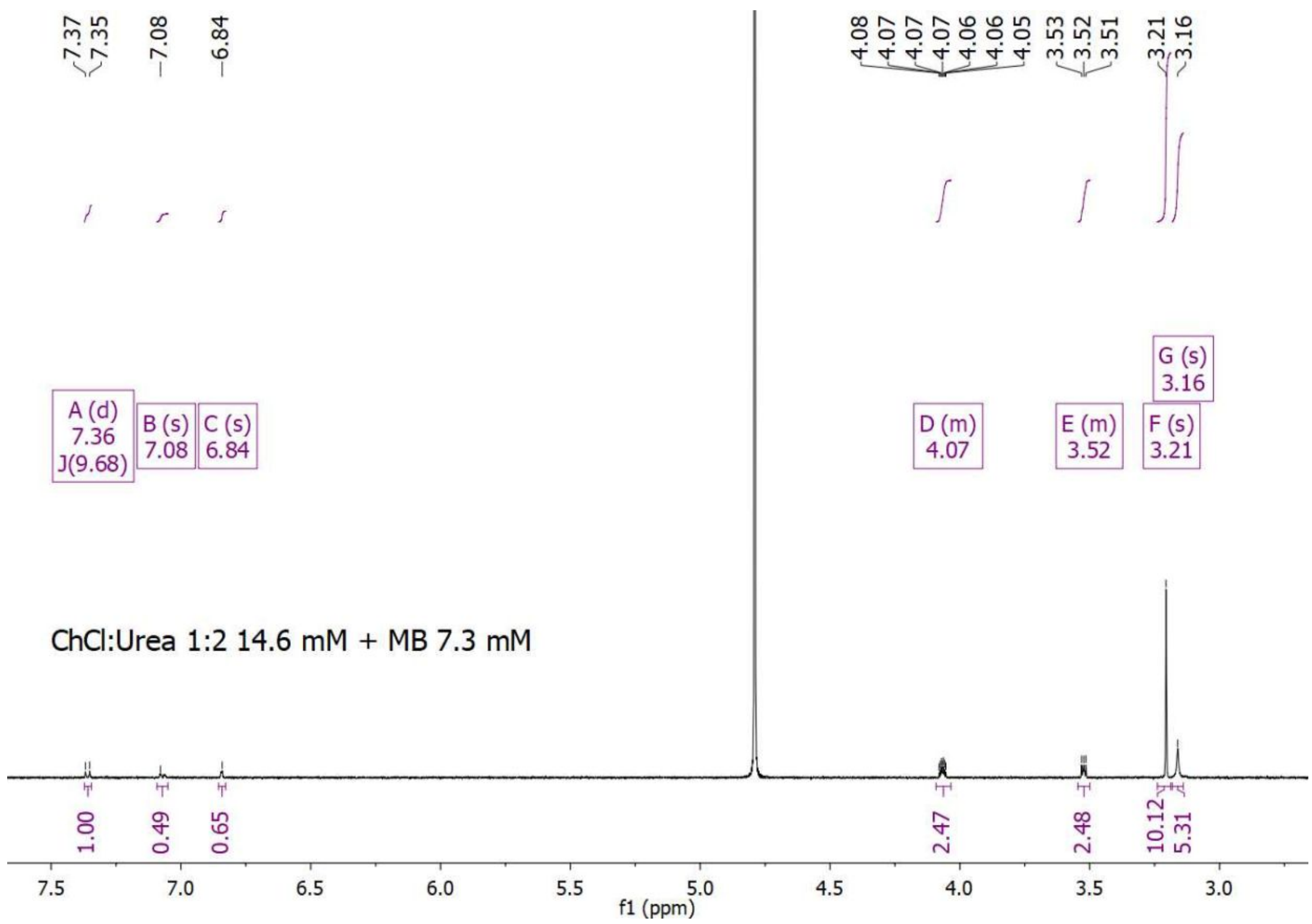


**Fig S16** $^{1}$H NMR spectrum of ChCl:Urea 1:2 14.6 mM + MB 7.3 mM ($D_2O$, 600 MHz, 25 °C).

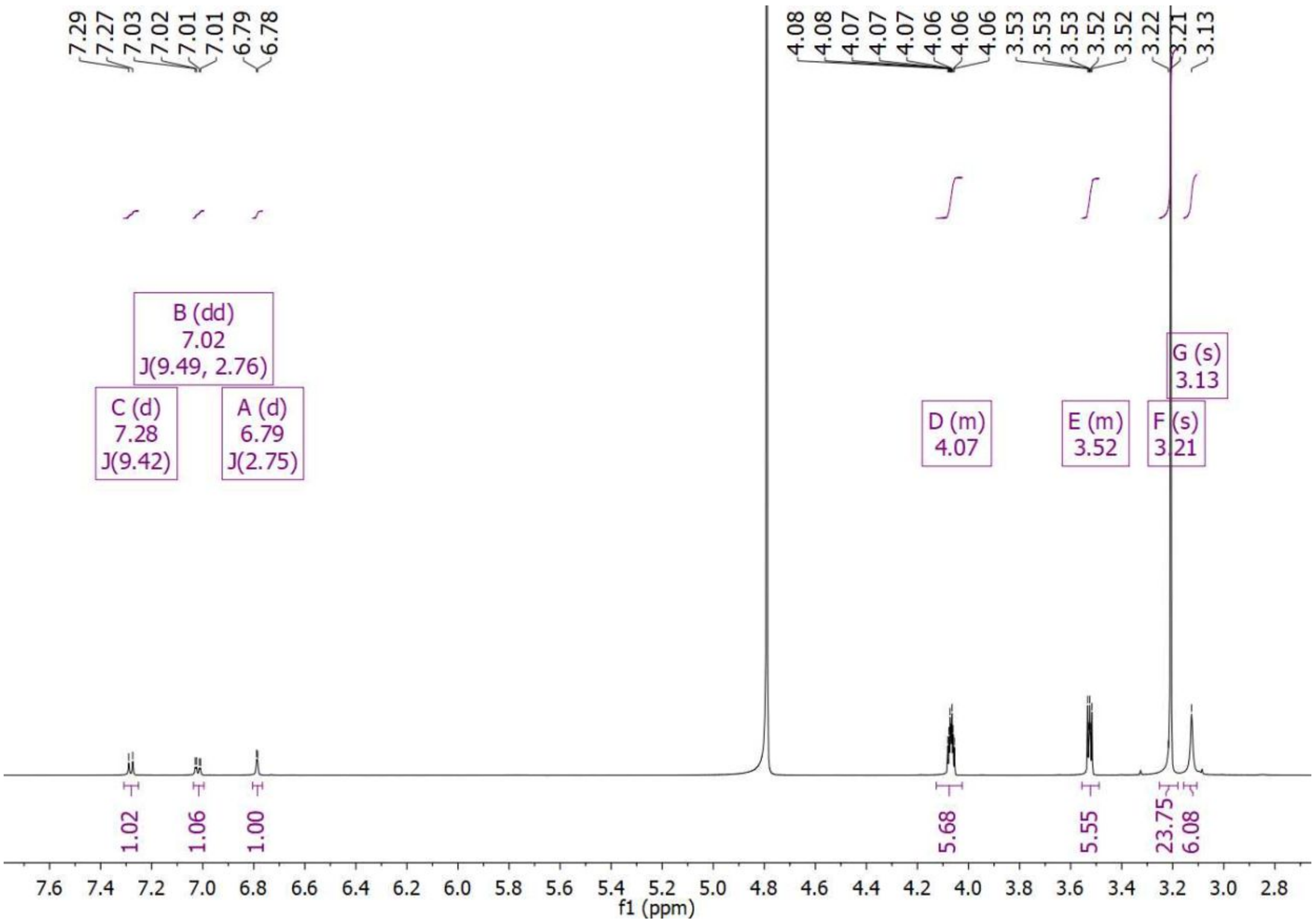


**Fig S17** $^{1}H$ NMR spectrum of ChCl:Urea 1:2 29.2 mM + MB 7.3 mM ($D_2O$, 600 MHz, 25 °C)

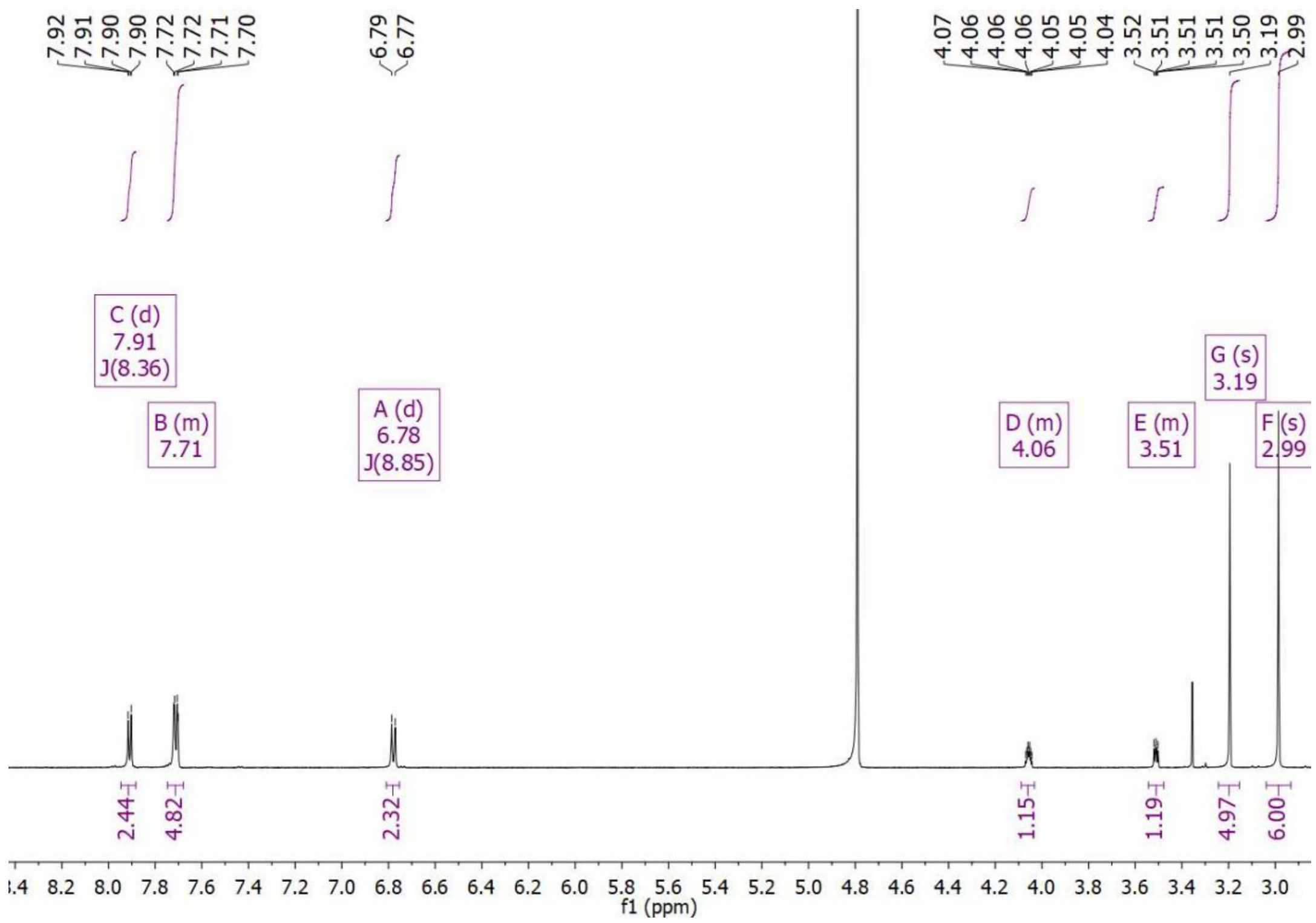


**Fig S18** $^{1}H$ NMR spectrum of ChCl:Urea 1:2 3.65 mM + MO 7.3 mM ($D_2O$, 600 MHz, 25 °C)

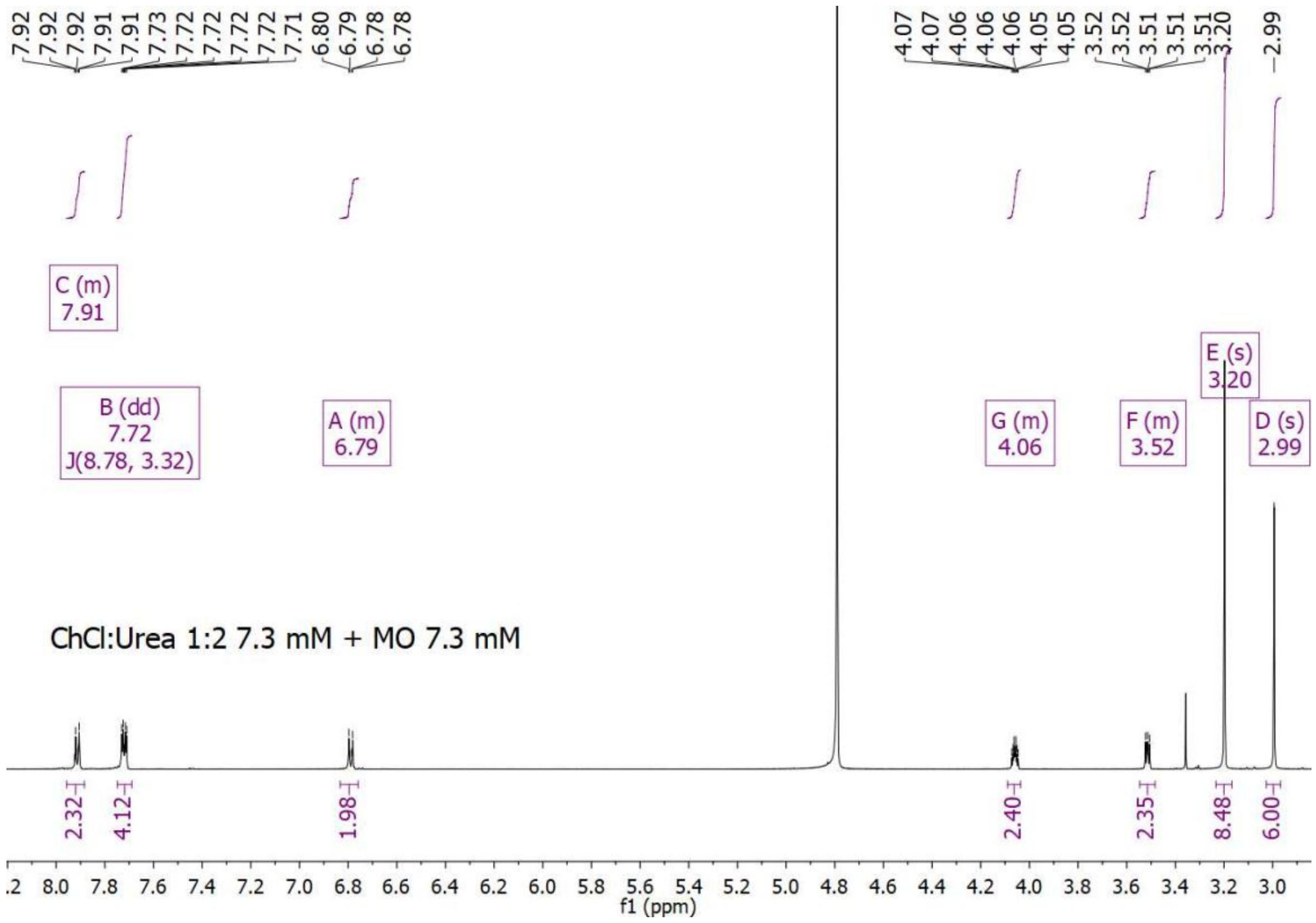


**Fig S20** $^{1}$H NMR spectrum of ChCl:Urea 1:2 7.3 mM + MO 7.3 mM ($D_2O$, 600 MHz, 25 °C).

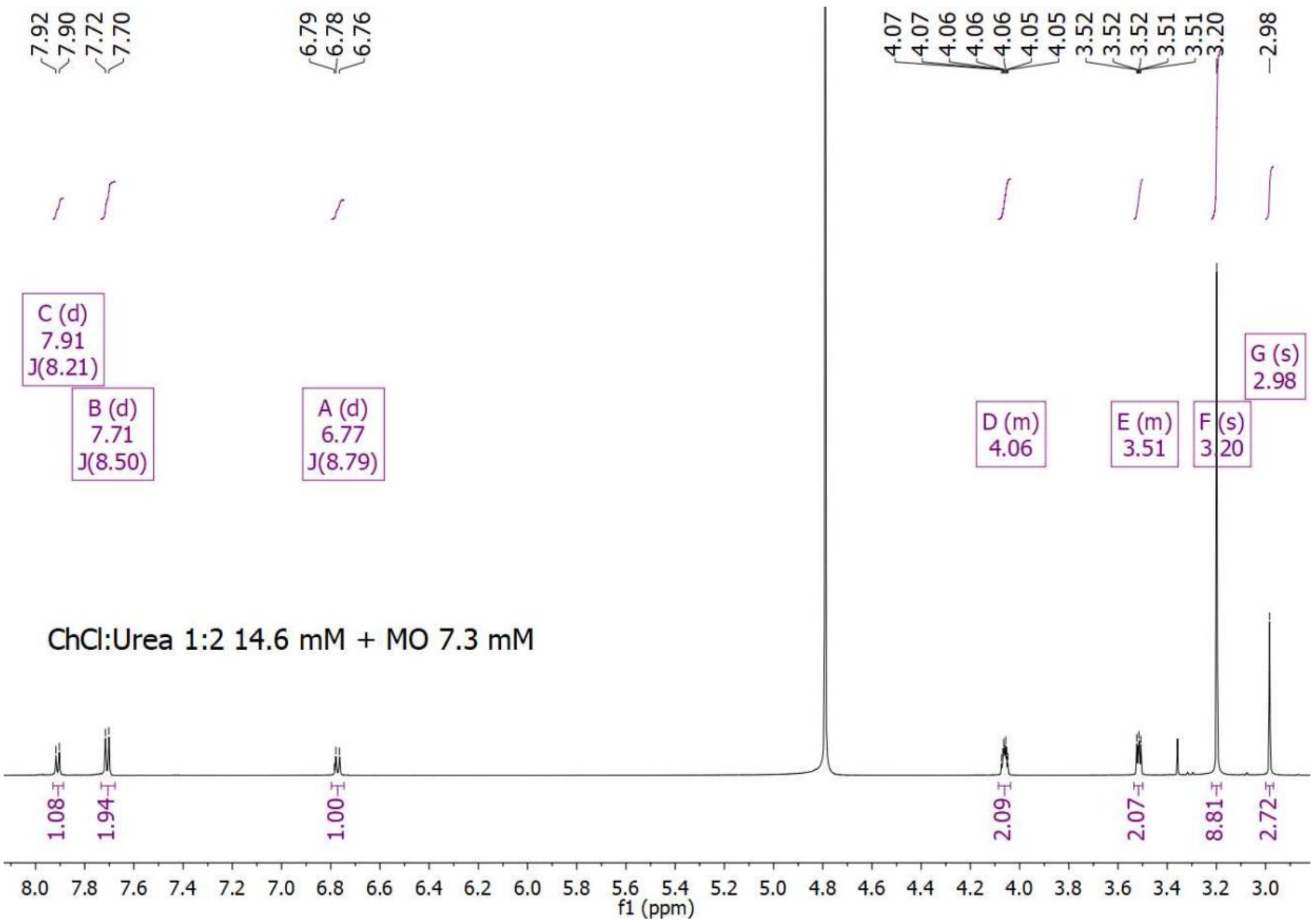


**Fig S21** $^{1}$H NMR spectrum of ChCl:Urea 1:2 14.6 mM + MO 7.3 mM ($D_2O$, 600 MHz, 25 °C).

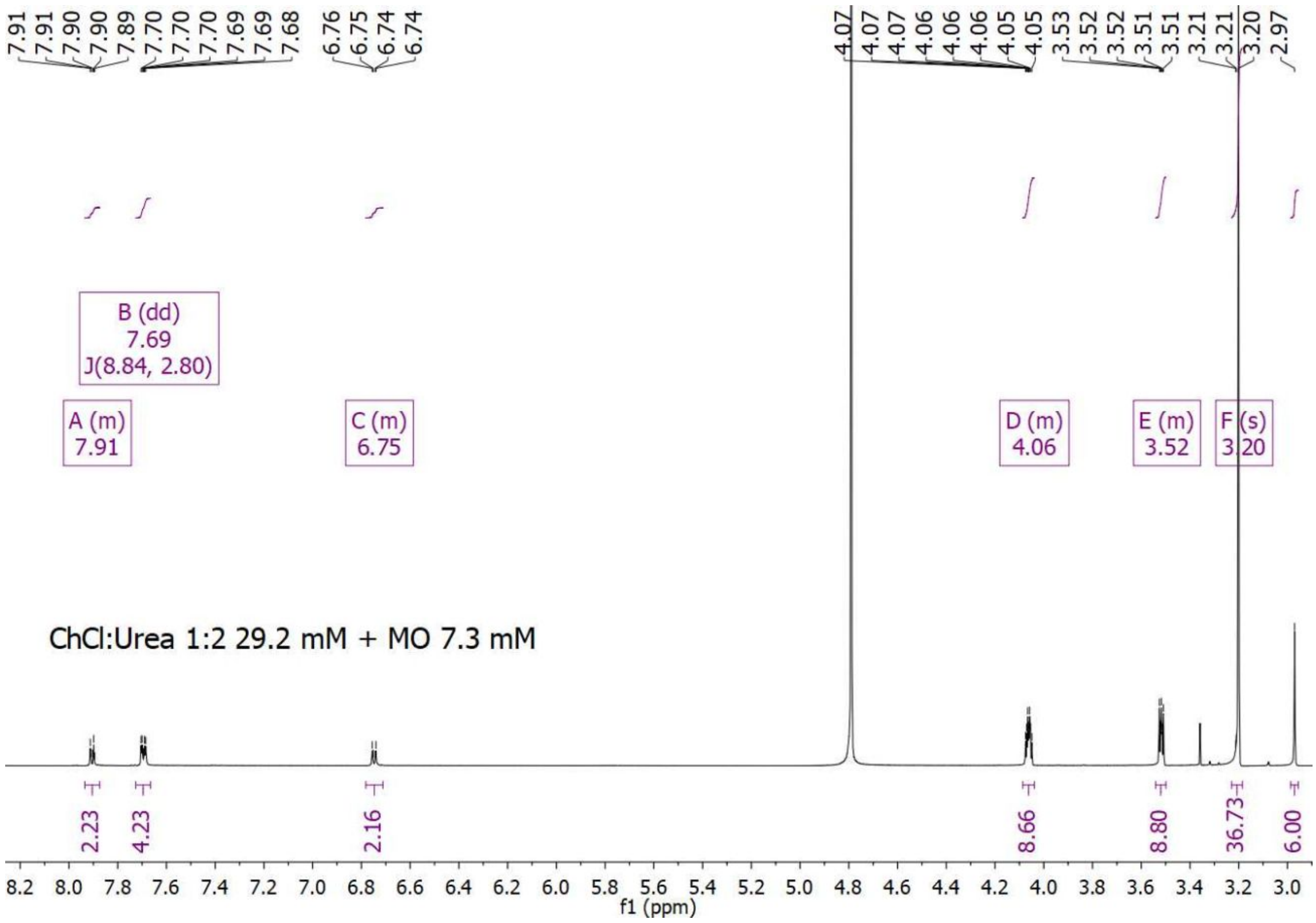


**Fig S22** $^1$H NMR spectrum of ChCl:Urea 1:2 29.2 mM + MO 7.3 mM ($D_2O$, 600 MHz, 25 °C).

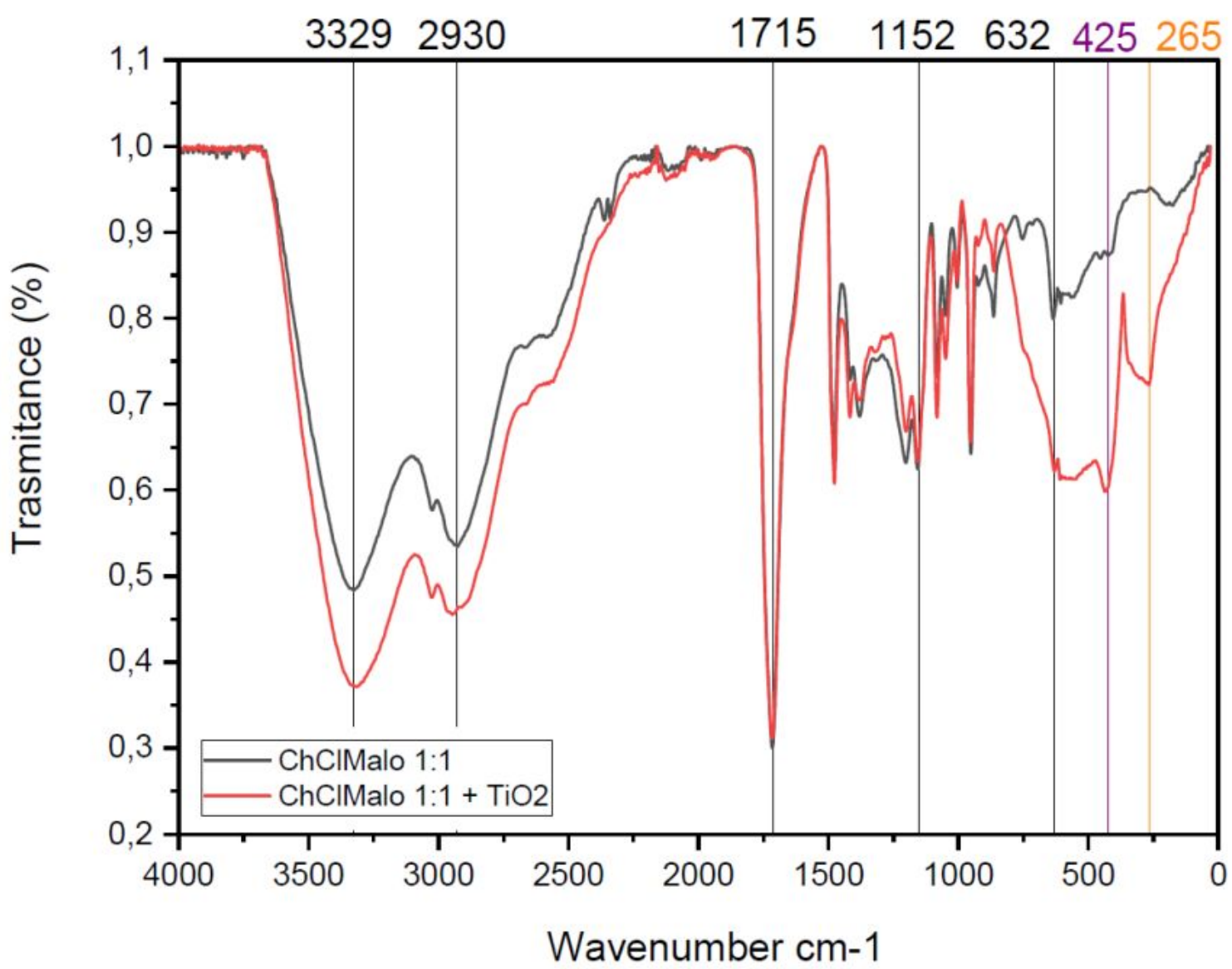


**Fig S23** FT-IR spectrum of ChCl:Malonic Acid 1:1 vs ChCl:Malonic Acid 1:1 + $TiO_2$

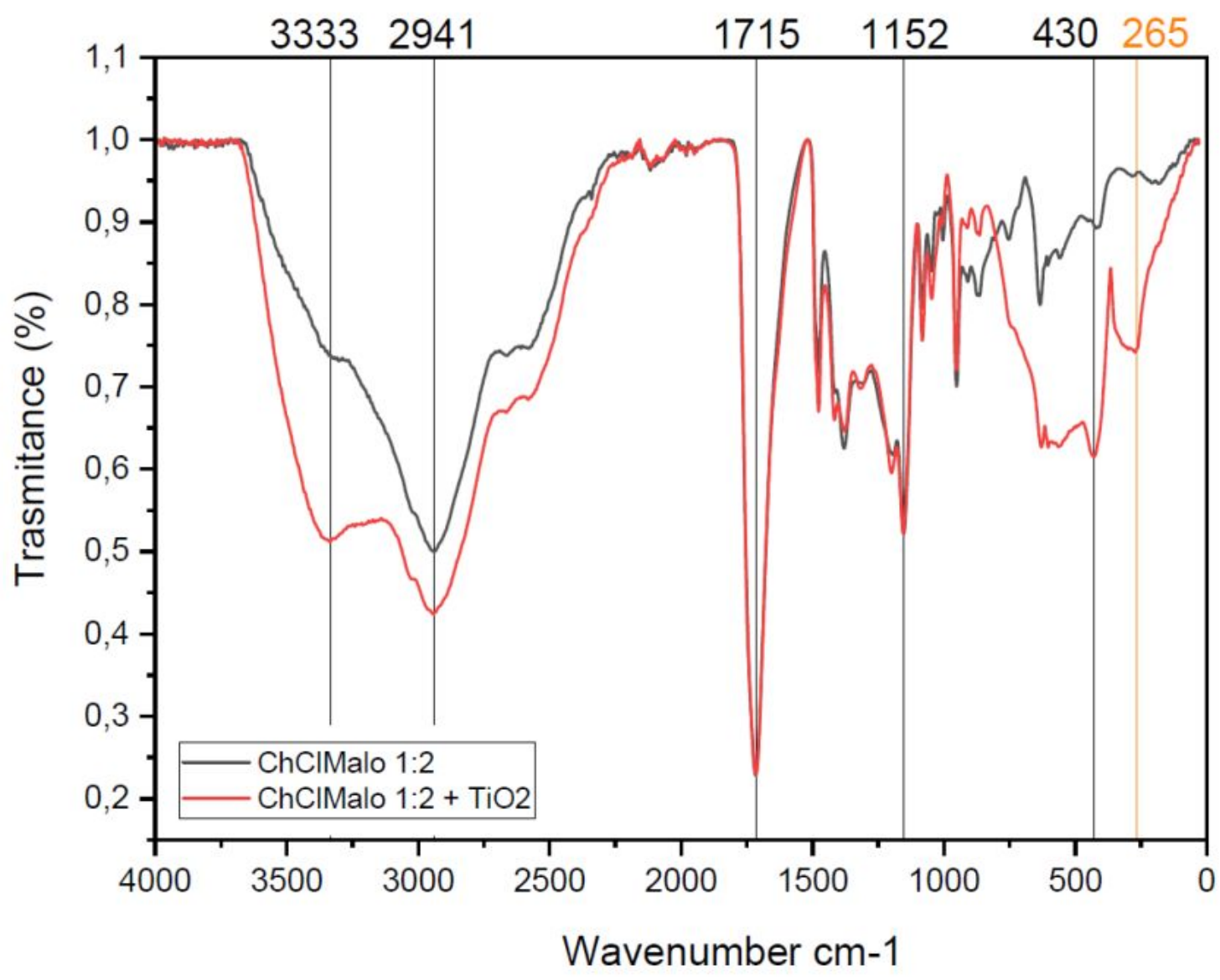


**Fig S24** FT-IR spectrum of ChCl:Malonic Acid 1:2 vs ChCl:Malonic Acid 1:2 + $TiO_2$

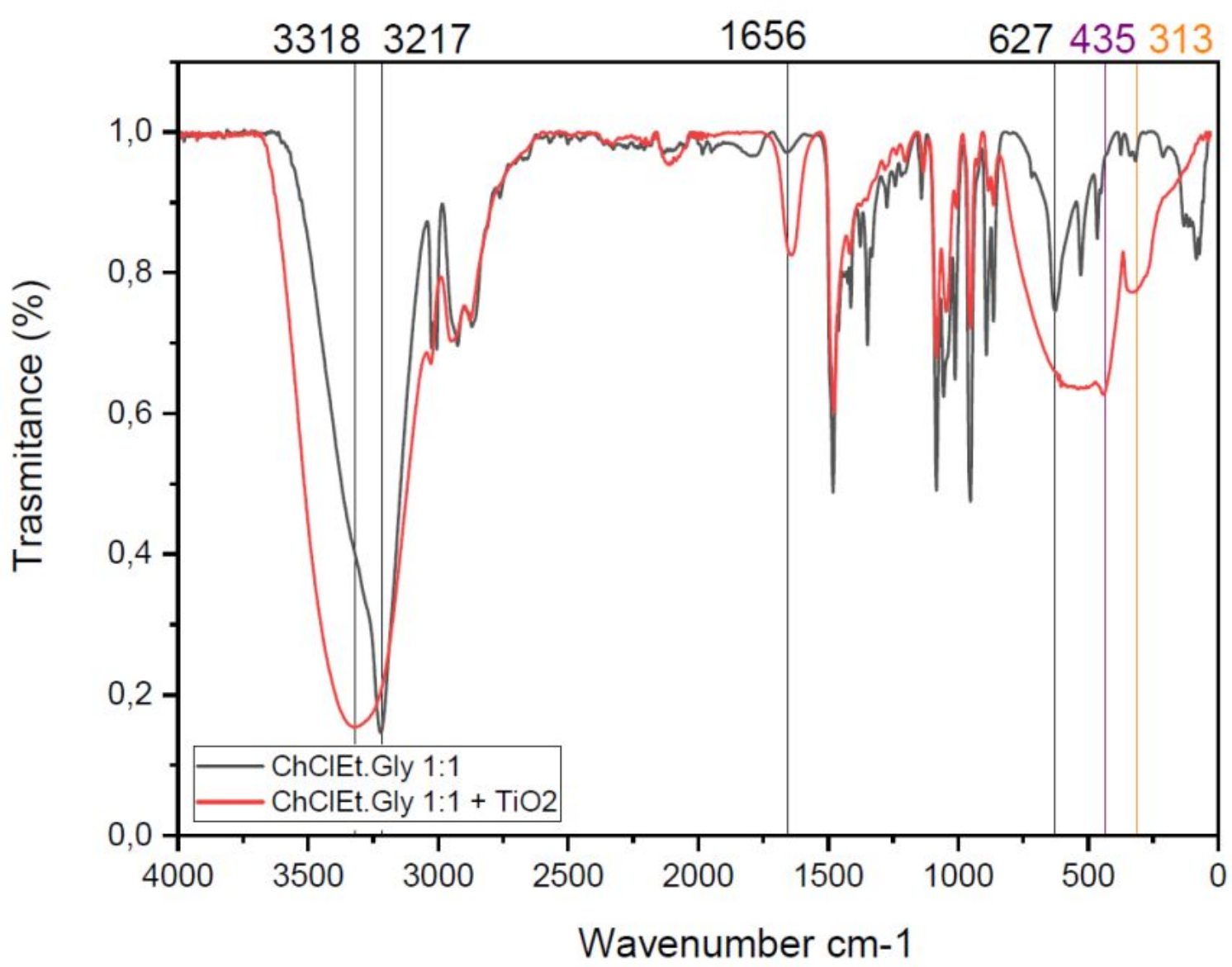


**Fig S25** FT-IR spectrum of ChCl:Ethylene Glycol 1:1 vs ChCl: Ethylene Glycol 1:1 + $TiO_2$

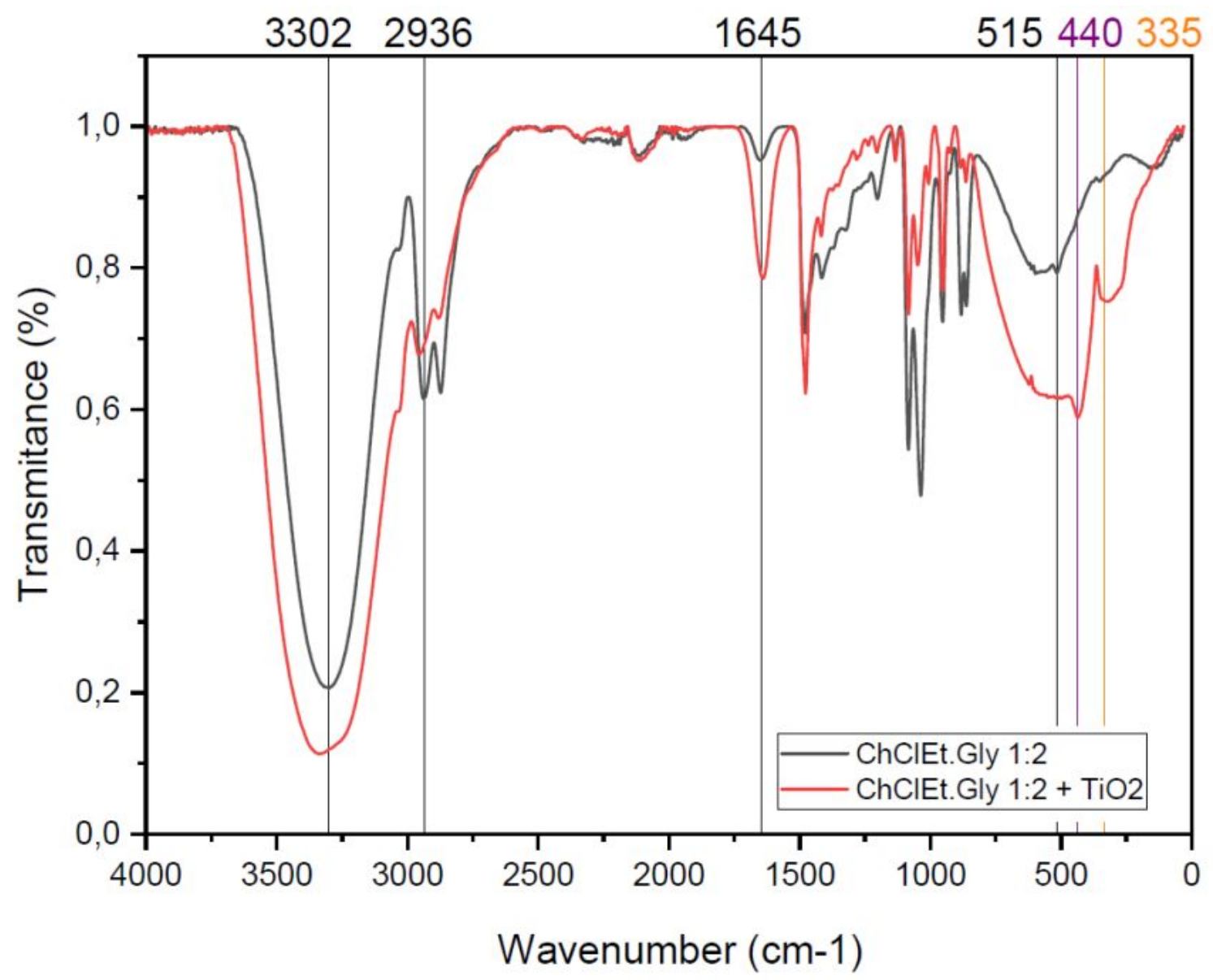


**Fig S26** FT-IR spectrum of ChCl:Ethylene Glycol 1:2 vs ChCl: Ethylene Glycol 1:2 + $TiO_2$

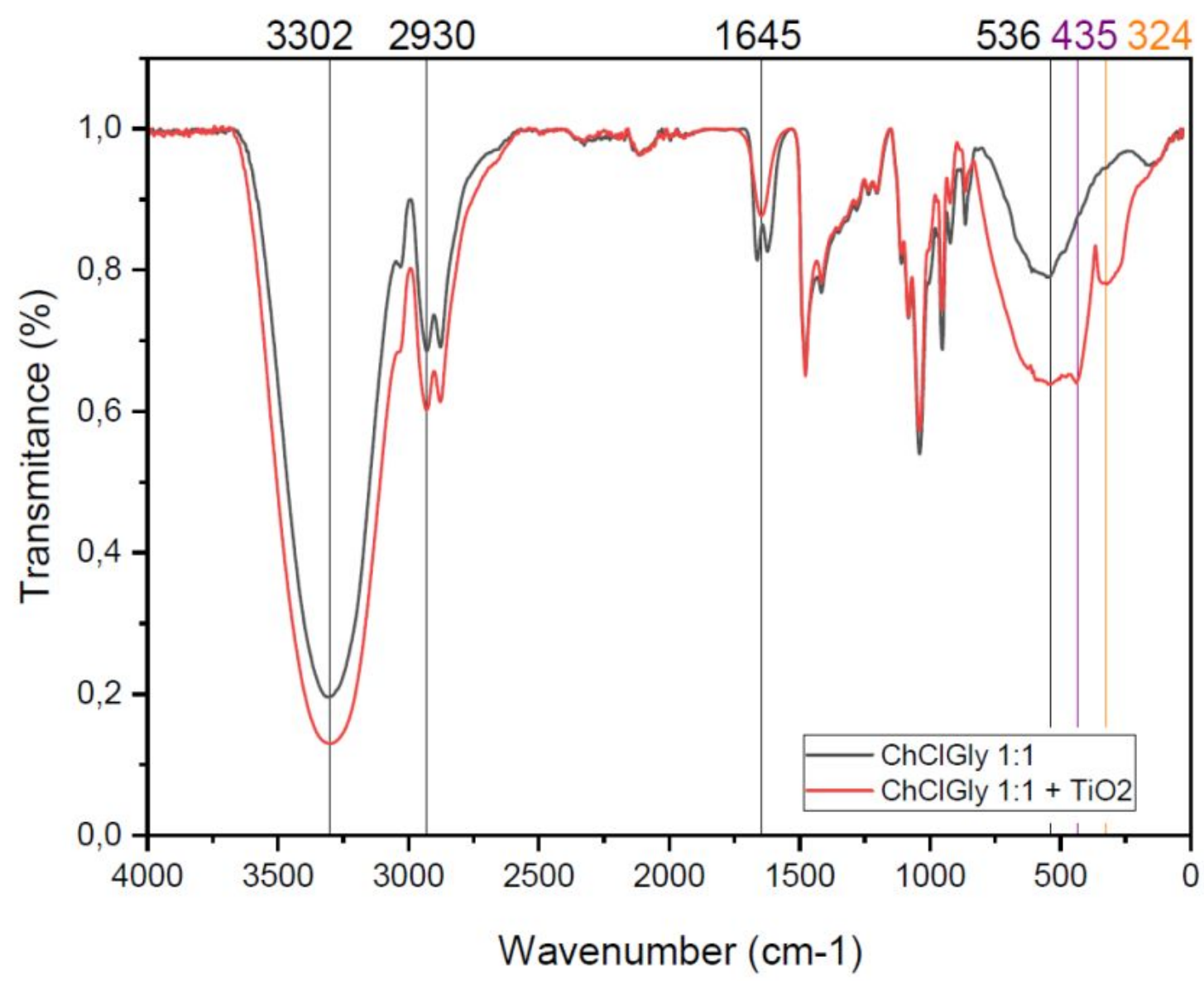


**Fig S27** FT-IR spectrum of ChCl:Glycerol 1:1 vs ChCl:Glycerol 1:1 + $TiO_2$

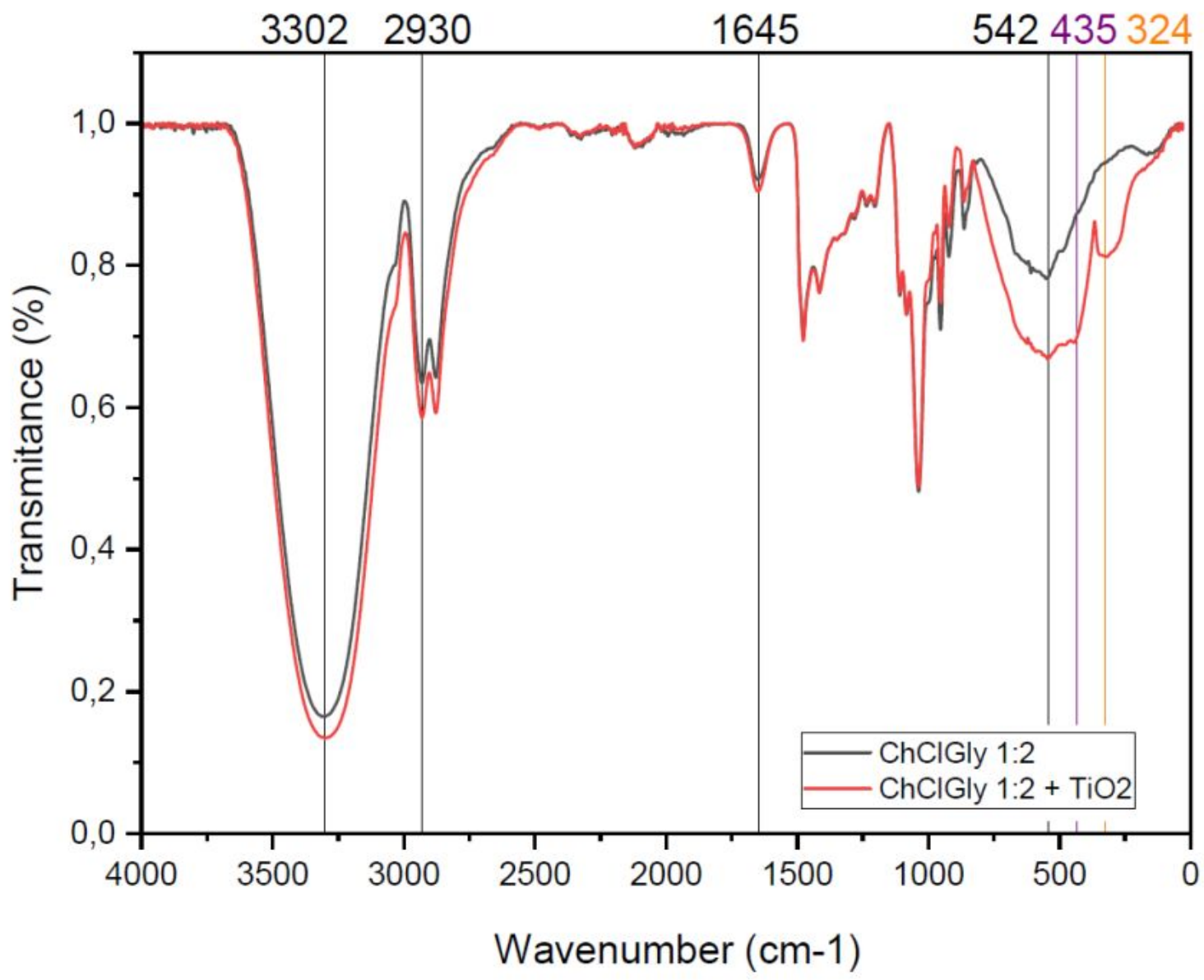


**Fig S28** FT-IR spectrum of ChCl:Glycerol 1:2 vs ChCl:Glycerol 1:2 + $TiO_2$

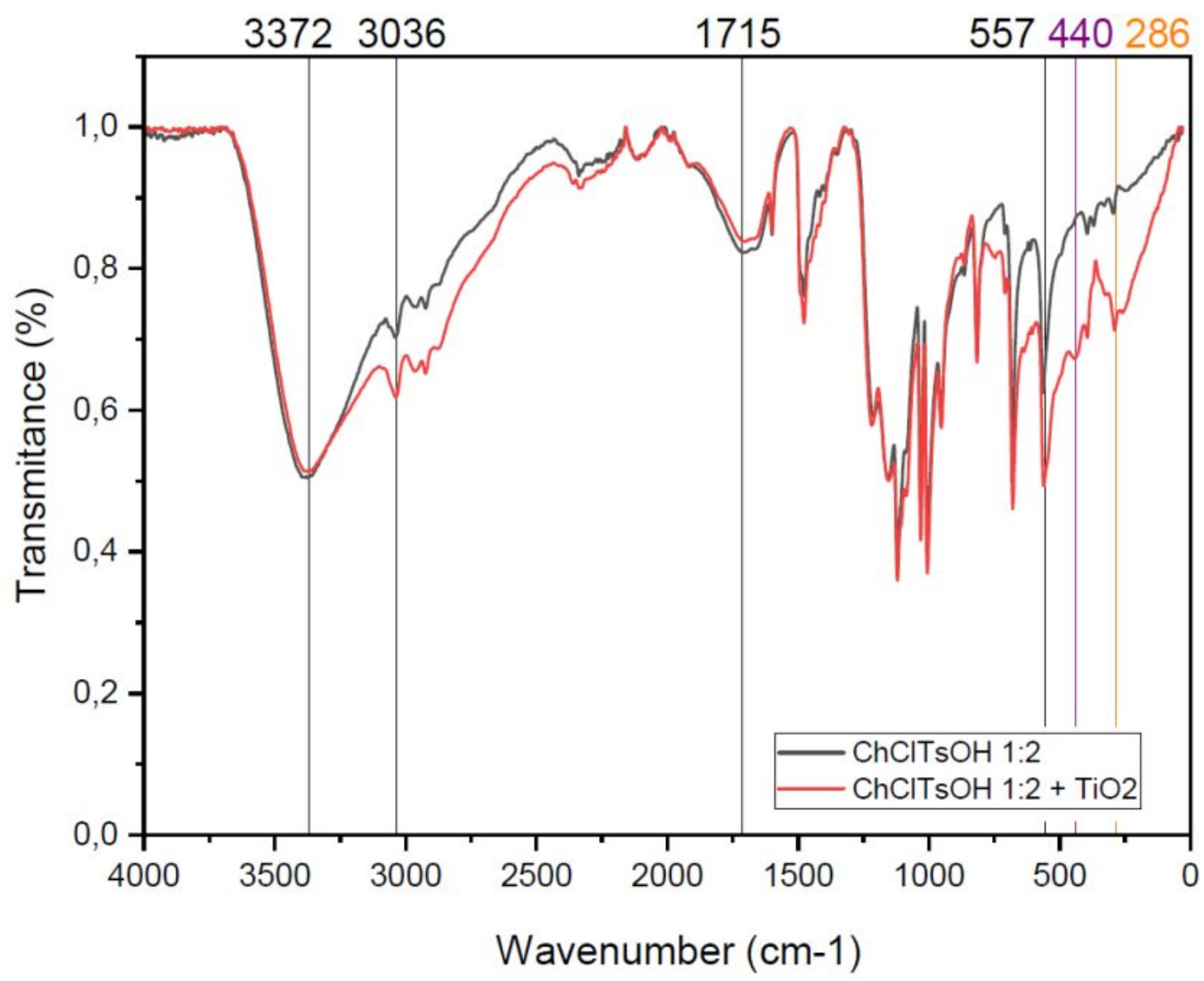


**Fig S29** FT-IR spectrum of ChCl:Toluenesulfonic Acid 1:2 vs ChCl:Toluenesulfonic Acid 1:2 + $TiO_2$

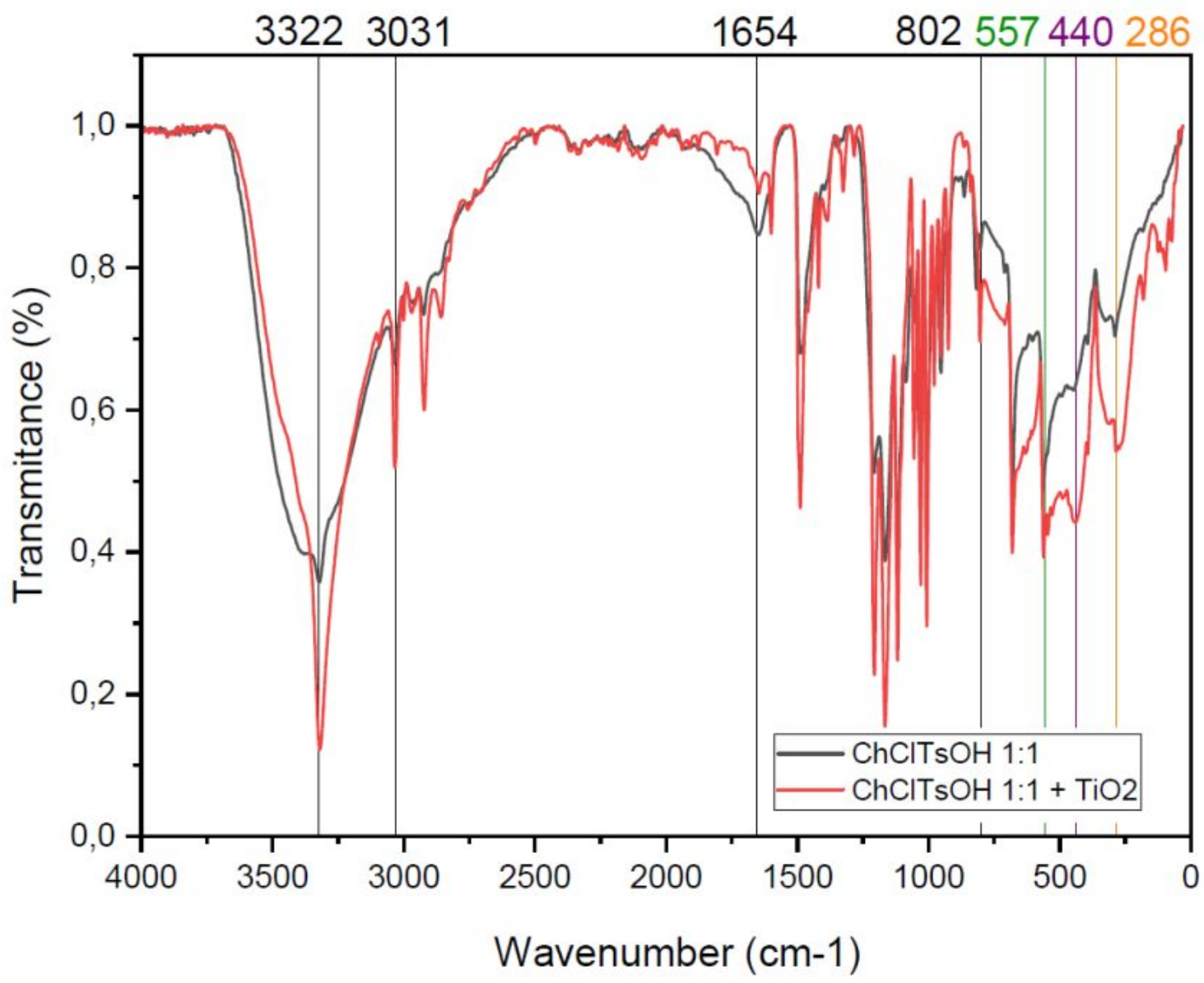


**Fig S30** FT-IR spectrum of ChCl:Toluenesulfonic Acid 1:1 vs ChCl:Toluenesulfonic Acid 1:1 + $TiO_2$

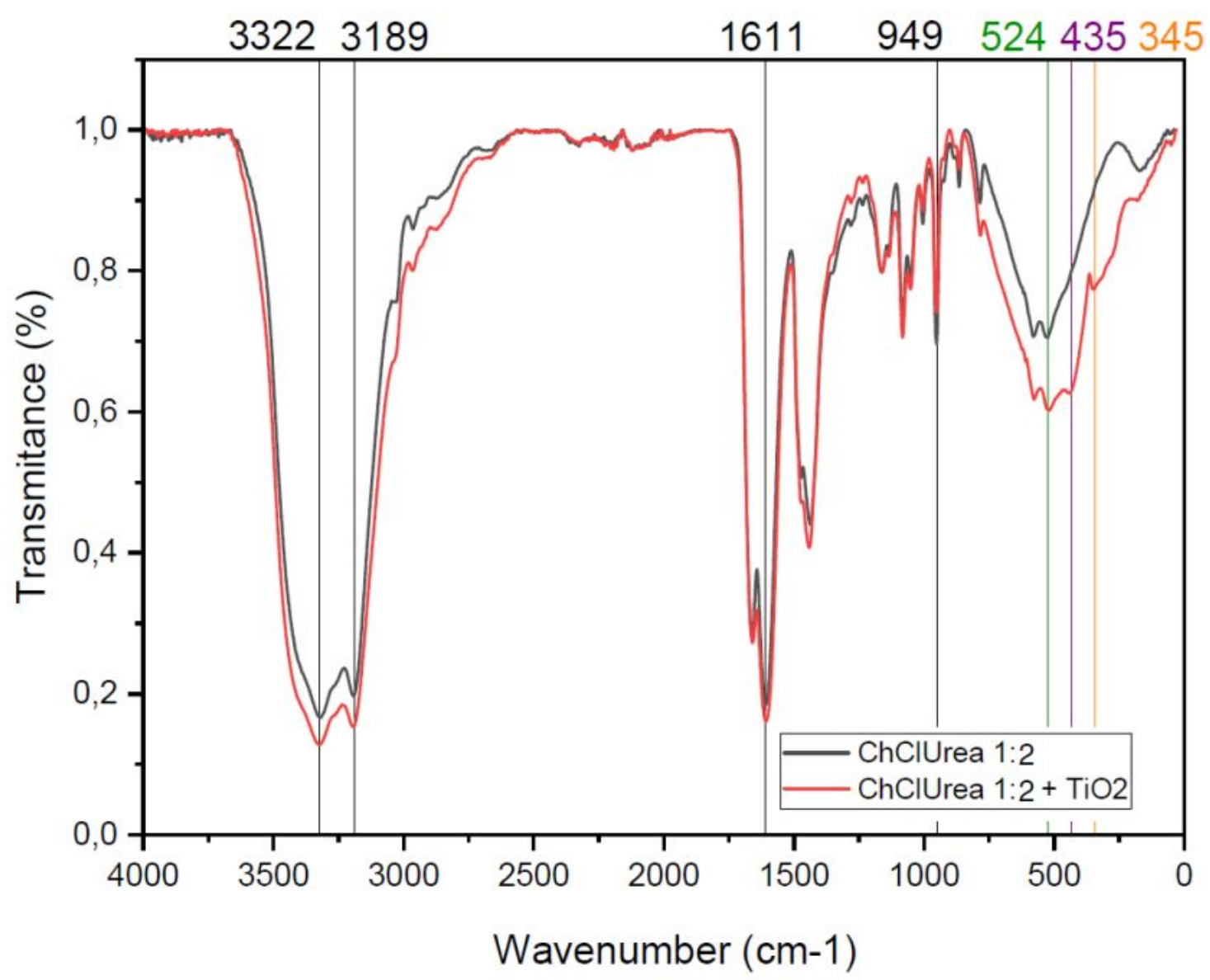


**Fig S31** FT-IR spectrum of ChCl:Urea 1:2 vs ChCl:Urea 1:2 + $TiO_2$

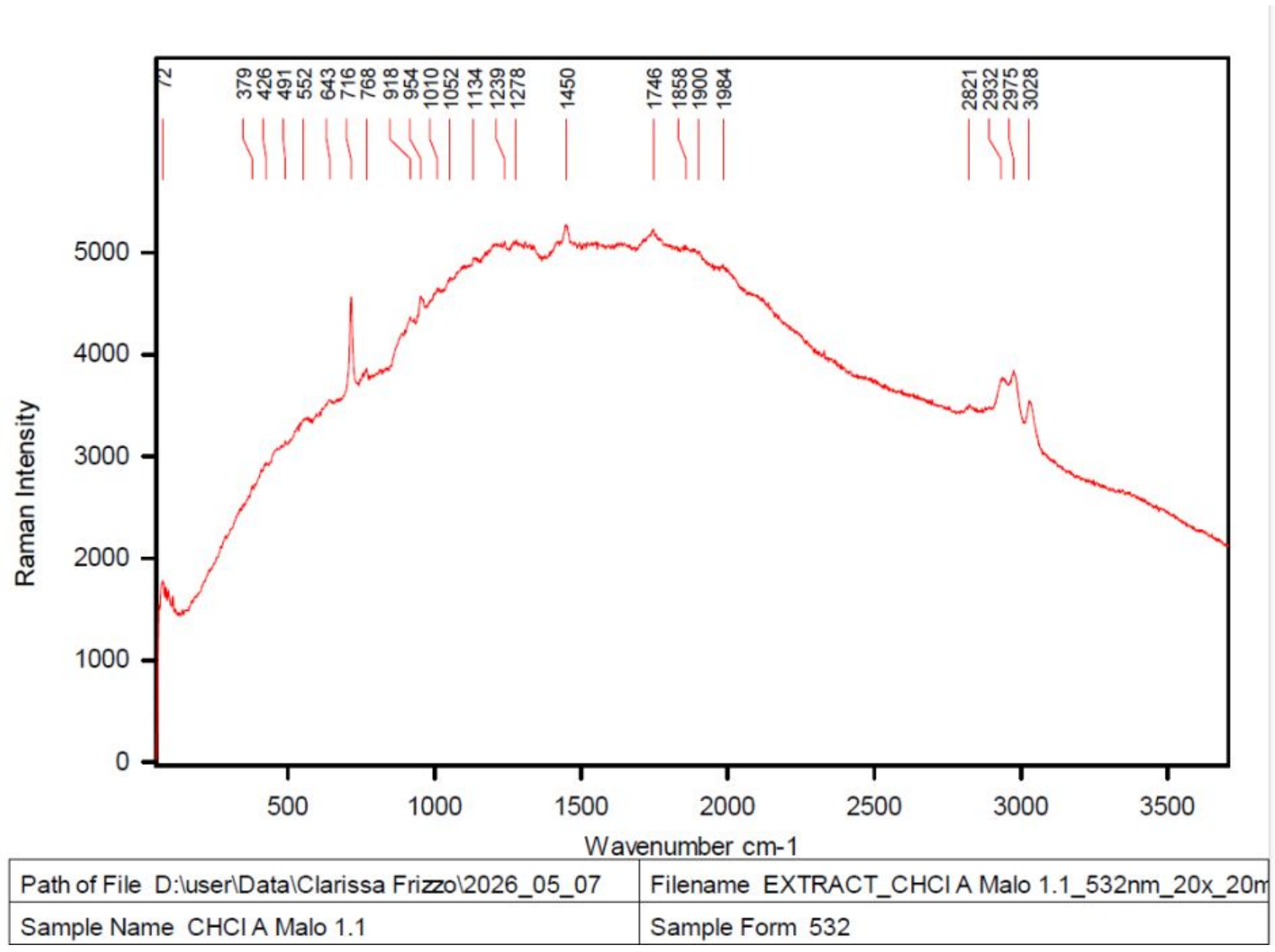


**Fig S32** RAMAN spectrum of pure ChCl:Malonic Acid 1:1

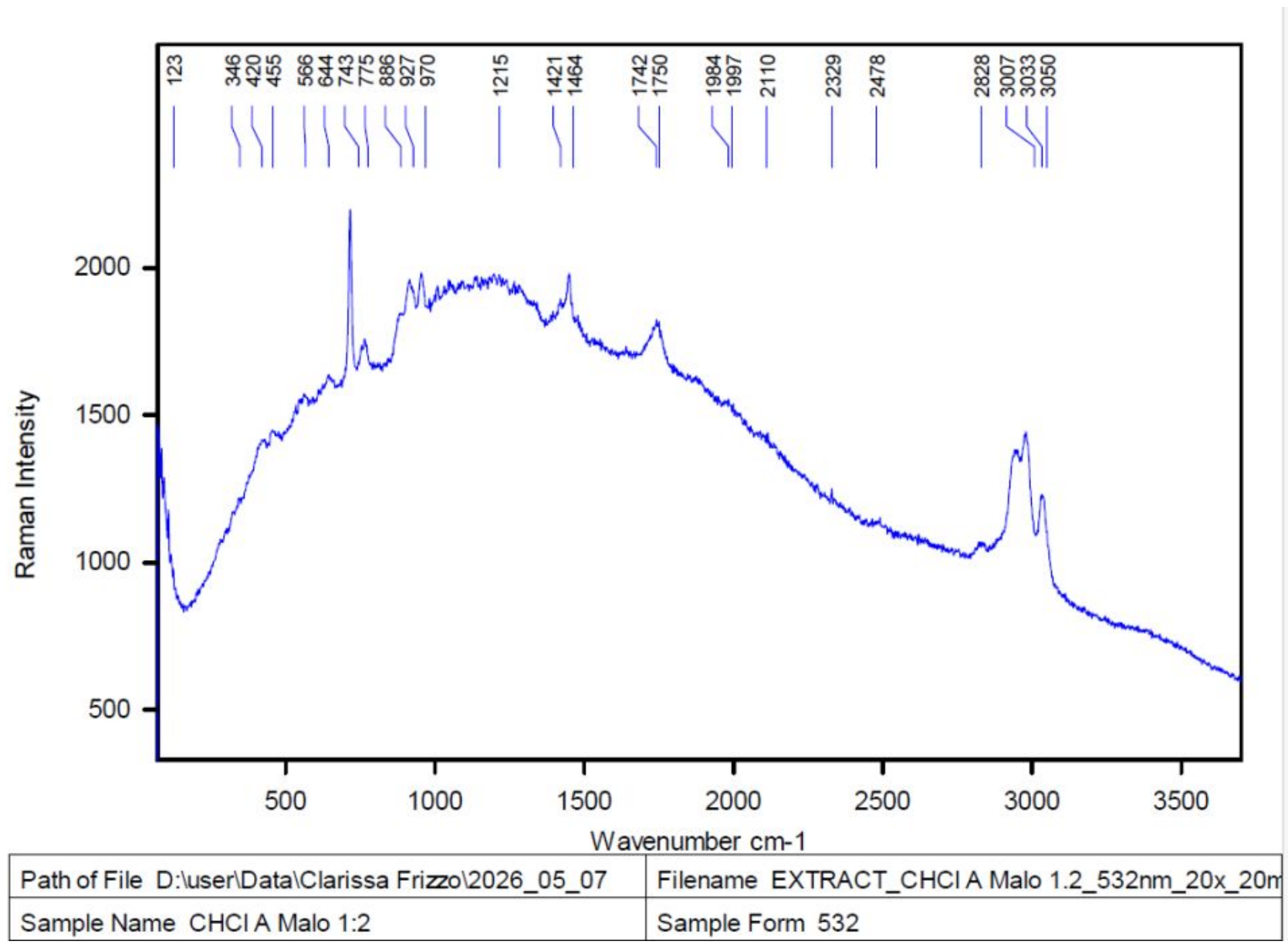


**Fig S33** RAMAN of pure ChCl:Malonic Acid 1:2

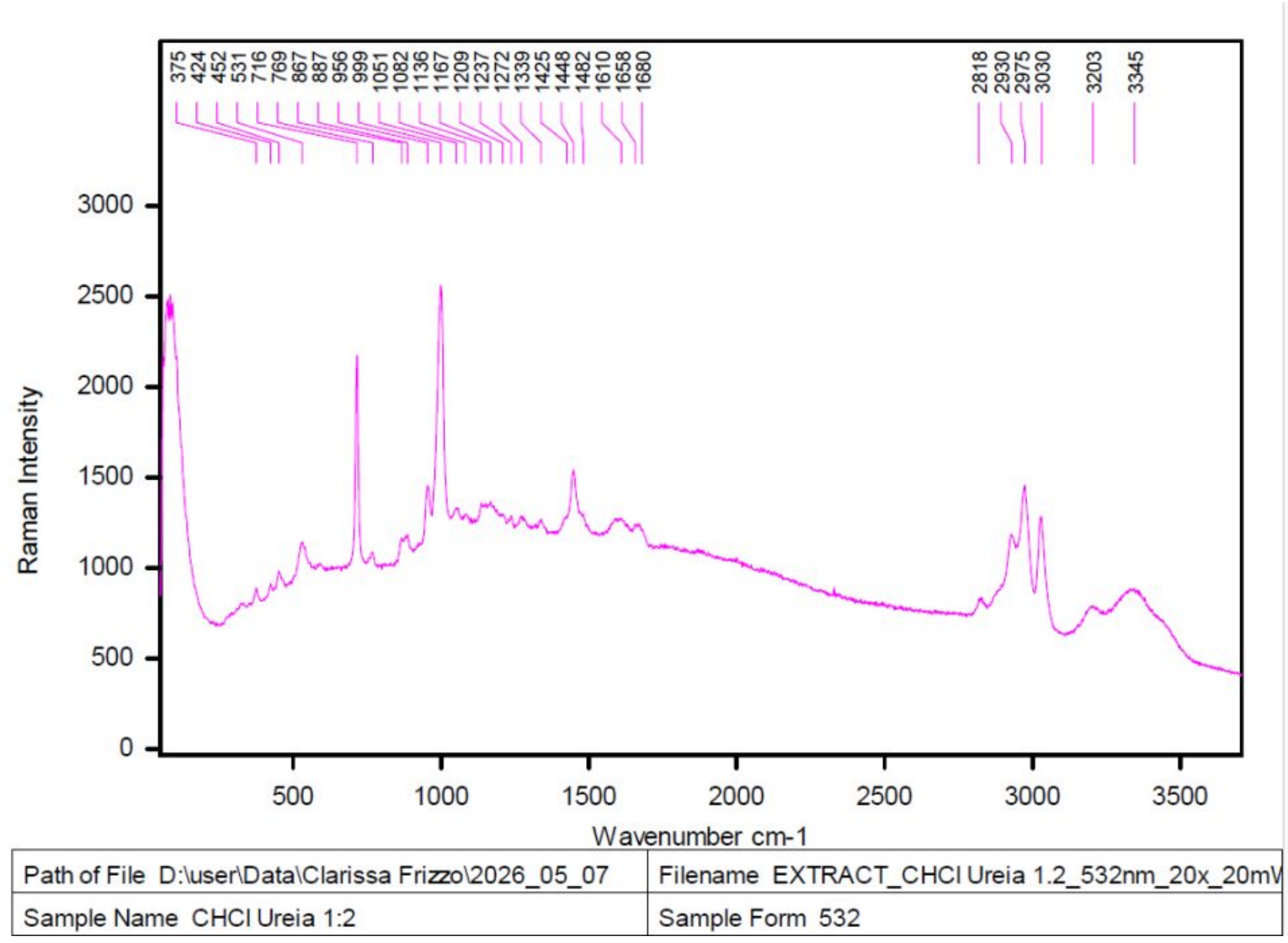


**Fig S34** RAMAN spectrum of pure ChCl:Urea 1:2

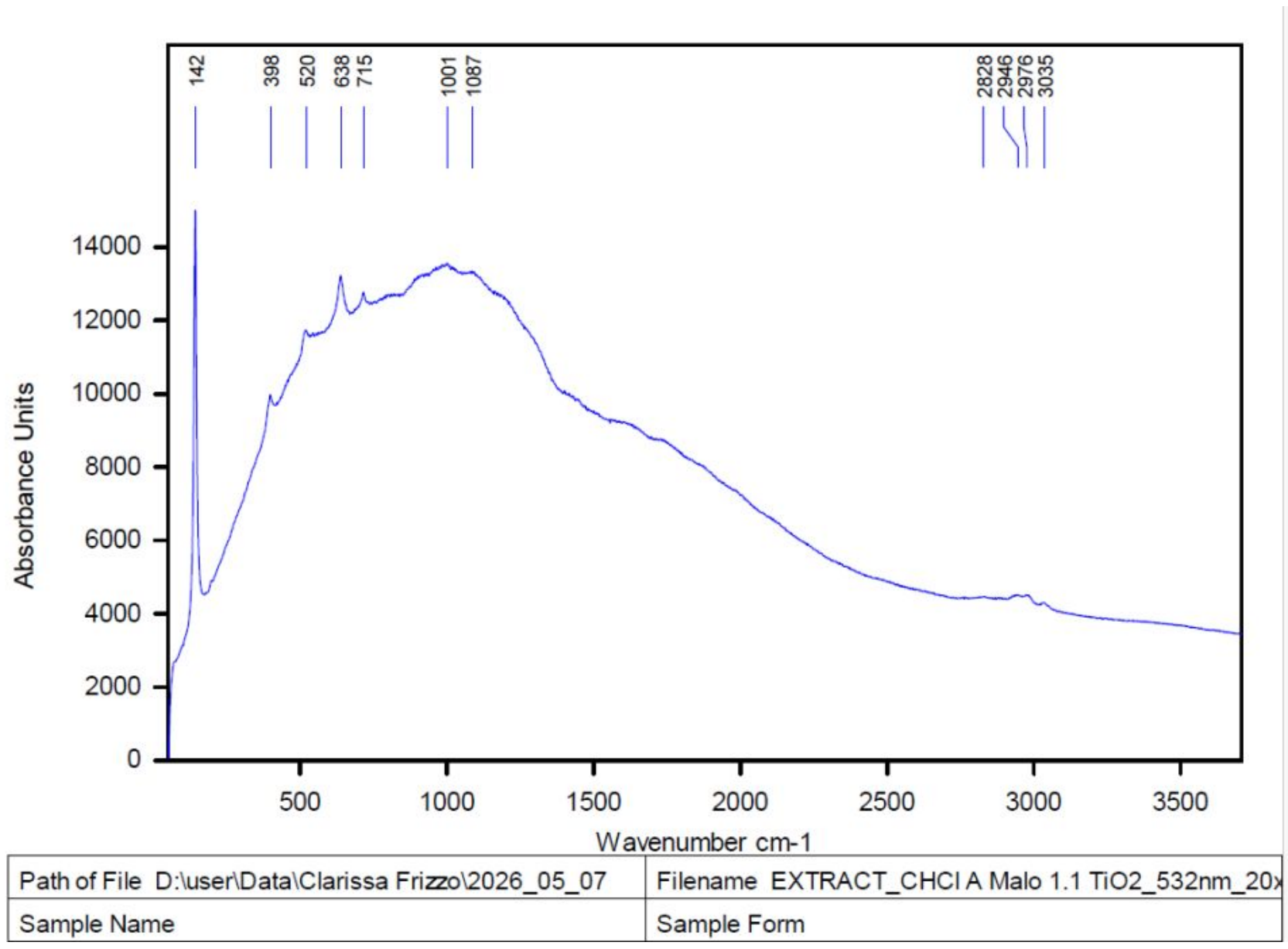


**Fig S35** RAMAN spectrum of ChCl:Malonic Acid 1:1 + $TiO_2$

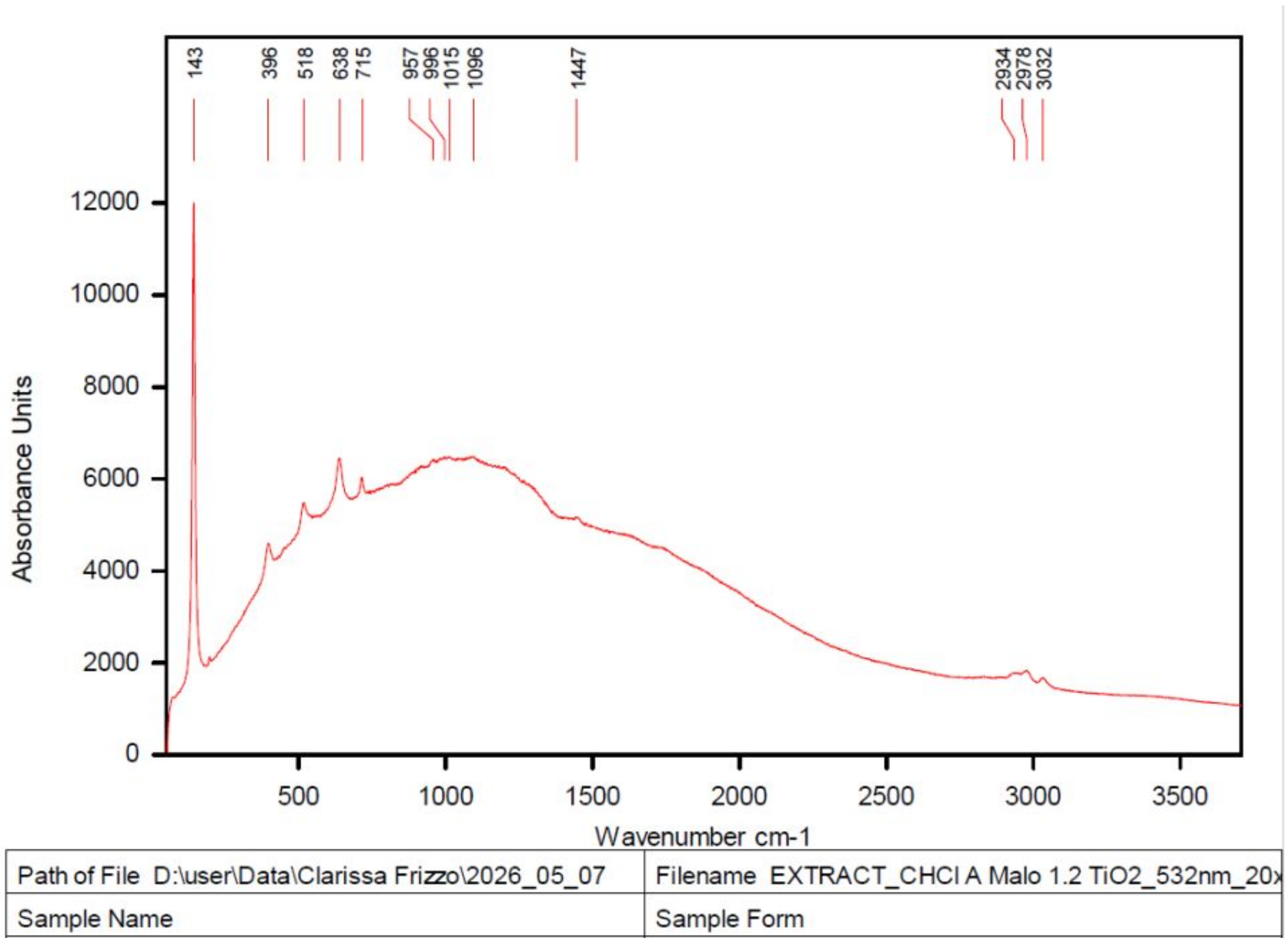


**Fig S36** RAMAN spectrum of ChCl:Malonic Acid 1:2 + $TiO_2$

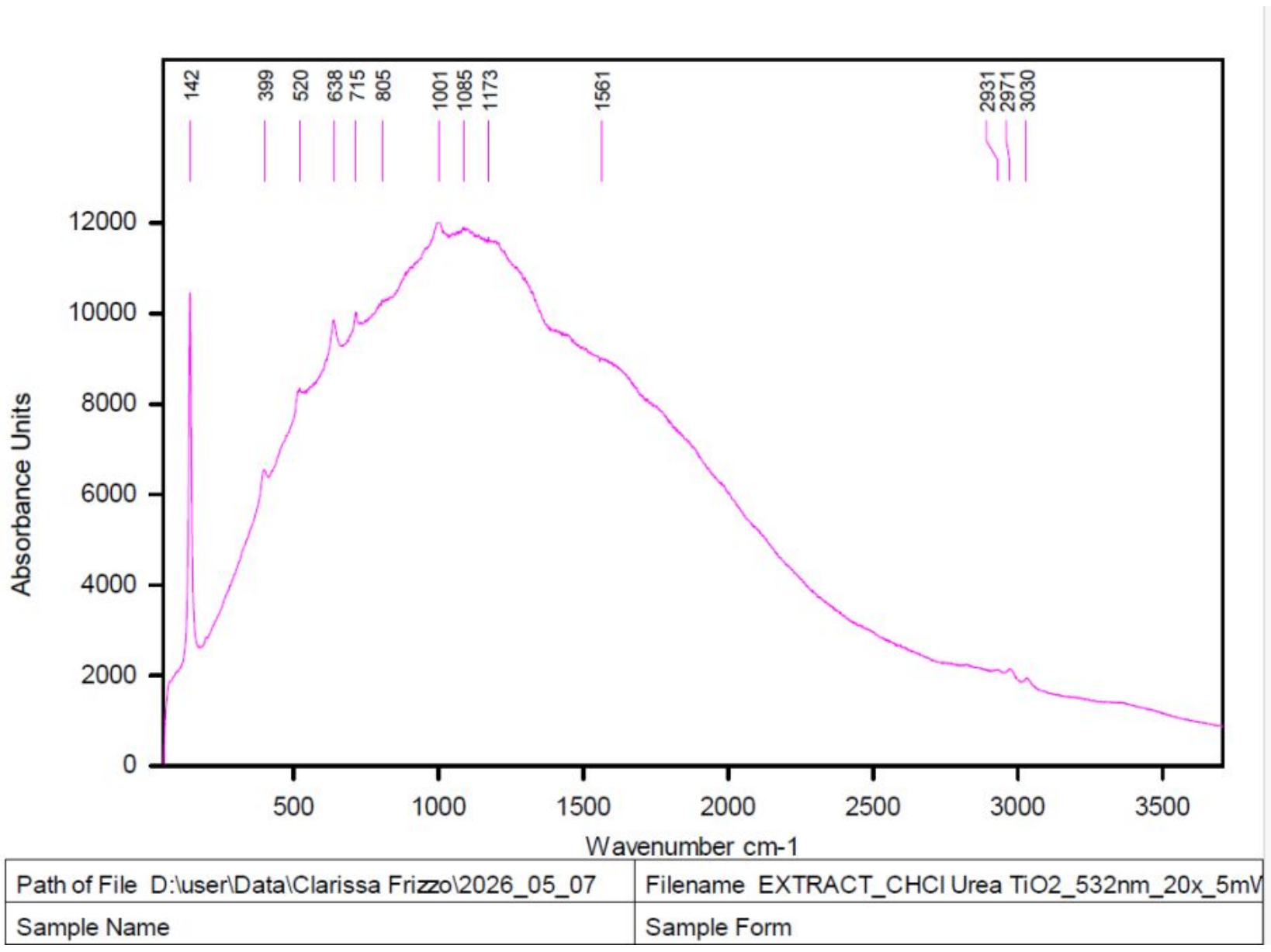


**Fig S37** RAMAN spectrum of ChCl:Urea 1:2 + $TiO_2$

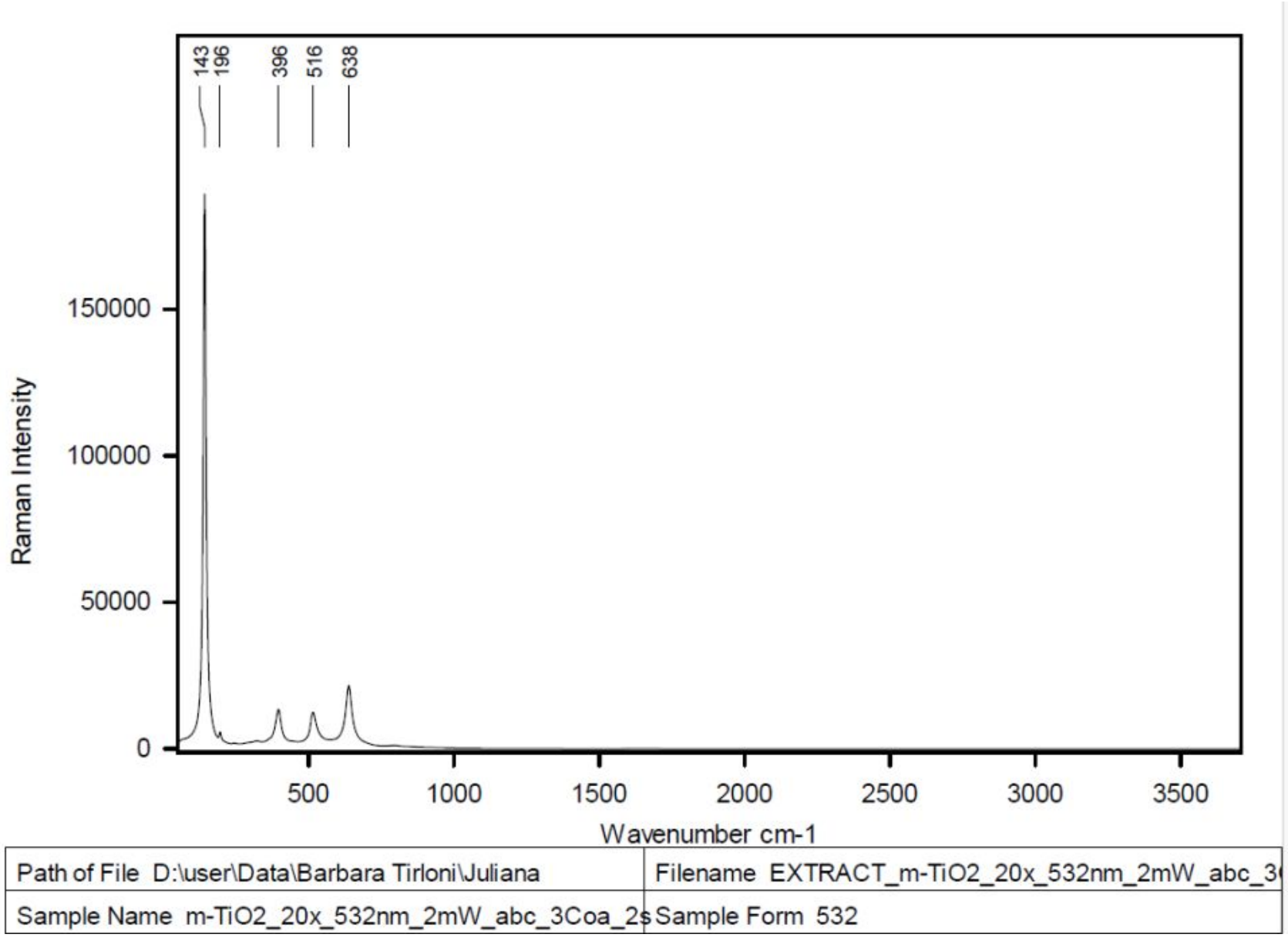


**Fig S38** RAMAN spectrum of pure $TiO_2$

**Table S1** Zeta Potential analysis for DES-$TiO_2$ samples.

| Sample | Zeta Potential (mV) |
|---|---|
| ChCl:Malonic Acid 1:1 + $TiO_2$ | 0.005926 ± 6.57 |
| ChCl:Malonic Acid 1:2 + $TiO_2$ | -0.2505 ± 8.46 |
| ChCl:Ethylene Glycol 1:1 + $TiO_2$ | -1.372 ± 5.79 |
| ChCl:Ethylene Glycol 1:2 + $TiO_2$ | -0.6378 ± 4.55 |
| ChCl:Glycerol 1:1 + $TiO_2$ | -8.81 ± 5.26 |
| ChCl:Glycerol 1:2 + $TiO_2$ | -5.814 ± 5.46 |
| ChCl:Toluenesulfonic Acid 1:1 + $TiO_2$ | -0.3299 ± 3.86 |
| ChCl:Toluenesulfonic Acid 1:2 + $TiO_2$ | 1.501 ± 38.03 |
| ChCl:Urea 1:2 + $TiO_2$ | 0.6047 ± 7.14 |
| Pure $TiO_2$ | -0.07431 ± 4.55 |
